# Multiscale Physicochemical Response of Lignocellulosic *Carludovica palmata* Fibers to Controlled Drying and Alkaline Hydrogen Peroxide Treatment

**DOI:** 10.3390/polym18202507

**Published:** 2026-10-13

**Authors:** Manuel Ayala-Chauvin, Sebastian Ojeda

**Affiliations:** Centro de Investigación en Ciencias Humanas y de la Educación (CICHE), Facultad de Ciencias de la Educación, Universidad Tecnológica Indoamérica, Ambato 180103, Ecuador; wojeda@indoamerica.edu.ec

**Keywords:** natural polymers, lignocellulosic fibers, cellulose, lignin, *Carludovica palmata*, cultural heritage, colorimetry, fractal analysis, thermal stability

## Abstract

Processing can alter the optical, structural, spectroscopic, morphological, and thermal characteristics of lignocellulosic fibers in different ways. Five measured states of *Carludovica palmata* were compared: an artisan-processed reference (PJ-0), water-conditioned fibers dried for 1 h at 84 and 104 °C (SPJ-84 and SPJ-104), and SPJ-104 fibers treated with two nominal alkaline hydrogen peroxide formulations (BPJ-3% and BPJ-6%). Relative image-based color analysis, X-ray diffraction (XRD), ATR-FTIR spectroscopy, scanning electron microscopy (SEM) coupled with fractal, multifractal, orientation, and spatial-texture analyses, and TGA/DTG were integrated within the same processing series. The BPJ-3% and BPJ-6% images showed similar measured appearance in the relative image-based color analysis, with a pairwise $\Delta E_{00}=1.7$, whereas their selected SEM fields differed in directional and boundary organization. The selected BPJ-3% field showed higher axial anisotropy (A=0.2835) than BPJ-6% (A=0.1183), while the selected BPJ-6% field showed $D_{B}=1.5570$ and a singularity-spectrum width of Δα=1.3444. In the individual XRD profiles, BPJ-3% showed higher apparent Scherrer and Segal descriptors than BPJ-6%, whereas the individual BPJ-6% TGA/DTG profile showed the highest principal degradation-rate temperature of the series ($T_{\max}=322.6$ °C). Because the principal physicochemical series contained one independent specimen per condition and one SEM, XRD, and TGA/DTG measurement set per condition, these numerical differences are interpreted as specimen-level observations rather than reproducible treatment-level effects. The study therefore provides an integrated multiscale description of the measured fiber states while explicitly delimiting the level of inference supported by the experimental design.

## 1. Introduction

Lignocellulosic fibers are multicomponent natural polymer systems in which semicrystalline cellulose microfibrils are embedded within a matrix of hemicellulose, lignin, extractives, water, and minor inorganic constituents. Their macroscopic behavior therefore arises from interactions across several structural scales rather than from cellulose content alone. This hierarchical organization helps explain why natural fibers are strongly influenced by their processing history and explains why complementary techniques such as X-ray diffraction, vibrational spectroscopy, microscopy, and thermal analysis are required to resolve structural, chemical, morphological, and thermal changes [1,2,3]. More broadly, the performance of lignocellulosic materials is governed by both the cellulose-rich framework and the surrounding non-cellulosic matrix, whose modification can alter accessibility, moisture response, interfacial behavior, and thermal stability [4,5].

Drying is an active part of this processing history rather than a neutral preparatory step. In cellulosic pulps, drying temperature has been associated with changes in reswelling, accessibility, and hornification [6,7]. Recent work on lignocellulosic substrates with different compositions further shows that hemicellulose and lignin influence the extent to which drying modifies cellulose accessibility and interfibrillar organization [8]. These observations are particularly relevant to heterogeneous plant fibers, because findings for purified cellulose cannot be applied directly to these materials. They also require a careful distinction between compact morphology in the dry state and irreversible hornification, which is demonstrated through changes in rewetting or reswelling behavior rather than by microscopy alone.

Chemical treatment provides an additional means of modifying the lignocellulosic matrix. Alkali can promote swelling, disrupt ester-linked and other interactions involving hemicellulose and lignin, and increase the accessibility of cellulose-rich domains, while peroxide introduces an oxidative environment capable of modifying chromophoric and aromatic structures. Reviews of natural-fiber modification and lignocellulosic pretreatment consistently emphasize that the resulting response depends on substrate composition, chemical loading, temperature, treatment time, and prior processing history [3,4,9]. Alkaline hydrogen peroxide has long been used to modify lignin-containing agricultural residues [10], and more recent studies have applied peroxide-based strategies to cellulose isolation from wheat straw and to the extraction and bleaching of almond-shell-derived fibers [11,12]. These treatments can improve optical appearance and alter the relative contribution of non-cellulosic domains, but they should not automatically be equated with cellulose purification or with an overall improvement in fiber quality.

The desired material state is therefore central to the interpretation of treatment severity. In pineapple leaf fibers, sequential organosolv pulping, peroxide bleaching, TEMPO-mediated oxidation, and microfluidization have been intentionally combined to obtain micro- and nanofibrillated cellulose [13]. In that context, extensive fibrillation is a desired endpoint. By contrast, when the functional objective is to retain a coherent lignocellulosic bundle, excessive delamination or fragmentation may be detrimental. Mechanical and structural studies of alkali-treated plant fibers similarly show that chemical modification must be evaluated in relation to the properties required for the intended application [3,14]. Increasing chemical severity should therefore be evaluated across optical, structural, morphological, and thermal endpoints rather than assumed to be inherently beneficial.

*Carludovica palmata*, commonly known as paja toquilla or jipijapa, provides a distinctive model for examining these relationships. Cellulosic fibers isolated from this species have previously been characterized in terms of morphology, crystallinity, and thermal behavior [15], and *Carludovica palmata*-derived cellulose has subsequently been incorporated into corn-starch-based biocomposite films [16]. In contrast, the present study examines artisan-processed lignocellulosic fibers that retain their hierarchical bundle architecture across additional water conditioning, controlled drying, and alkaline hydrogen peroxide treatment states. This study contributes an integrated multiscale characterization of these processing states by combining relative optical descriptors, quantitative SEM-based morphological and spatial-texture analysis, XRD, ATR-FTIR, and TGA/DTG within the same experimental series. This approach allows complementary optical, morphological, structural, spectroscopic, and thermal descriptors to be compared while the fiber architecture is retained, rather than after dedicated cellulose isolation, pulping, or nanofibrillation.

The starting material is itself a product of a specialized processing tradition. In the Barcelona community of Santa Elena, Ecuador, the cultivation and preparation of paja toquilla are closely linked to the production chain of the Ecuadorian toquilla straw hat, whose traditional weaving was inscribed in 2012 on UNESCO’s Representative List of the Intangible Cultural Heritage of Humanity [17]. The documented sequence includes harvesting, separation of the fibrous material from surrounding tissues, boiling, drying, and subsequent whitening or conditioning steps [17]. From a materials-science perspective, these operations are important because they establish the thermal, mechanical, chemical, and moisture history of the fiber before any laboratory treatment is applied.

**Figure 1:**
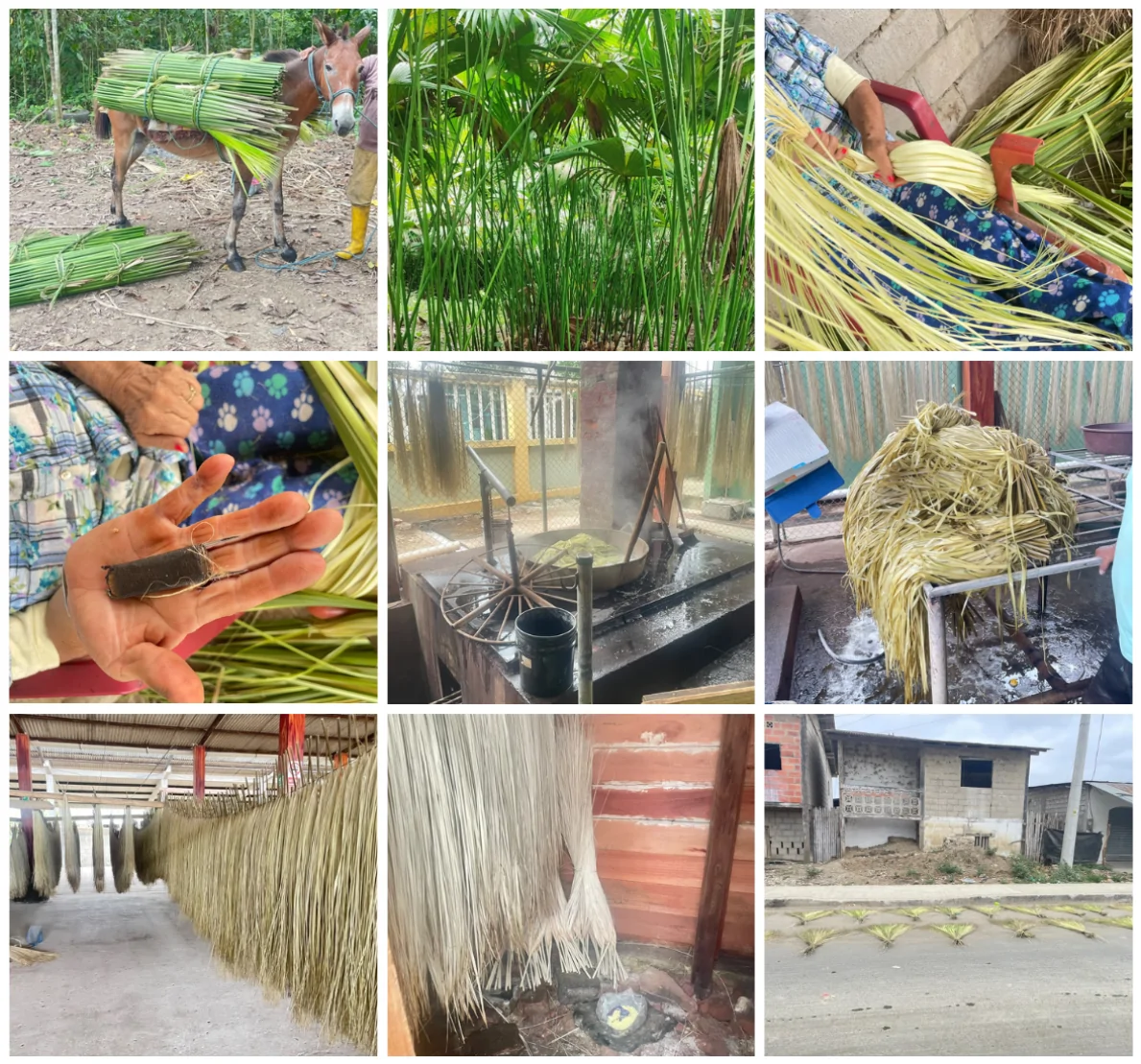
Field documentation of the traditional *Carludovica palmata* processing context in the Barcelona community, Santa Elena, Ecuador. The montage illustrates cultivation and transport of harvested plant material, manual fiber preparation and longitudinal subdivision, traditional tools, hydrothermal cooking, drainage, drying, conditioning, and handling of the resulting paja toquilla fibers.

Figure 1 documents the processing environment observed during fieldwork in the Barcelona community. The collage shows *Carludovica palmata* cultivation, transport of harvested material, manual fiber preparation, traditional tools, hydrothermal cooking, drainage, drying, and conditioning. This context establishes the technological provenance of the PJ-0 reference and clarifies that it represents an already processed lignocellulosic material rather than pristine plant tissue.

The traditional and laboratory processing routes must nevertheless be distinguished chemically. The artisanal sequence in Santa Elena may include sulfur-based sahumado as a whitening or conditioning operation, whereas the laboratory series examined here uses alkaline hydrogen peroxide. The laboratory treatment was not designed to reproduce sahumado; rather, it provides defined processing contrasts for comparing the measured fiber states within the limits of the experimental design. PJ-0 therefore serves as a technologically meaningful artisan-processed reference state rather than as untreated botanical material. Because all principal specimens originated from a single artisan source and batch, the present series provides a detailed characterization of the measured material states rather than a population-representative description of *Carludovica palmata* fibers across different plants, harvests, artisan batches, or processing locations.

Relative image-based color analysis and quantitative SEM image analysis address two complementary dimensions of this problem. Image-derived CIELAB coordinates describe relative differences in lightness, yellowness, chroma, and color separation within the analyzed image set, whereas SEM-based boundary, grayscale, lacunarity, multifractal, orientation, and spectral descriptors characterize how the observed microstructure is distributed across multiple spatial scales. These descriptors are not expected to produce identical numerical orderings: a selected field may exhibit high boundary complexity but weak directional organization, while specimen images with similar measured appearance may show different SEM morphologies. Their combined value lies in comparing complementary optical and morphological characteristics of the measured fiber states without treating agreement among descriptors as independent replication.

Accordingly, this study compares optical, structural, spectroscopic, morphological, and thermal descriptors across five measured processing states of *Carludovica palmata* and examines where complementary analytical techniques provide convergent or divergent descriptions of those states. The conditions were compared using relative image-based color analysis, X-ray diffraction (XRD), attenuated total reflectance Fourier-transform infrared spectroscopy (ATR-FTIR), scanning electron microscopy (SEM) coupled with fractal, multifractal, orientation, and spatial-texture analysis, and thermogravimetric/derivative thermogravimetric analysis (TGA/DTG). A separate operational fraction-recovery experiment provided context for the lignocellulosic character of the material without treating the recovered fractions as chemically pure constituents. The objective was to establish an integrated multiscale description of the measured fiber states while distinguishing specimen-level observations from within-image or model-fitting variability. The principal characterization series was not designed to estimate a population-level dose-response relationship or an optimal treatment concentration.

## 2. Materials and Methods

### 2.1. Material Provenance and Reagents

The reference material (PJ-0) was obtained from the Centro Artesanal de Procesamiento de Paja Toquilla “Barcelona”, Santa Elena, Ecuador, during fieldwork conducted on 22–25 July 2026. PJ-0 was an artisan-processed fiber rather than an untreated botanical control. The documented local sequence included manual preparation and longitudinal subdivision, hydrothermal cooking, drainage, and drying. Sulfur-based fumigation (sahumado) is used optionally within this processing context; however, its application to the specific PJ-0 batch could not be confirmed, and no matched sulfur-treated comparator was included in the experimental series.

NaOH, H_2_O_2_, HCl, and ethanol were supplied by AP Solutions EC (Quito, Ecuador). The peroxide stock was labeled 30%, and absolute ethanol was used for fraction recovery. Distilled water was used for all bleaching-bath preparations. Because the concentration basis of the commercial peroxide stock was not recorded, the BPJ sample codes are retained as nominal treatment labels and the bath formulations are reported explicitly below.

### 2.2. Processing Sequence and Sample Nomenclature

Fiber portions of 1.00 g were immersed in water at 54 °C for 1 h and subsequently dried for 1 h at either 84 or 104 °C, yielding SPJ-84 and SPJ-104, respectively. The SPJ labels refer to the prescribed drying temperatures, and the drying endpoint was defined by the 1 h treatment duration. No post-drying conditioning at controlled temperature and relative humidity was applied. Residual moisture was not independently measured.

SPJ-104 served as the common precursor for the alkaline peroxide series. Each treatment bath contained 1.60 g NaOH and either 4.0 or 8.0 mL of the peroxide stock labeled 30%, with distilled water added to a final volume of 40 mL. The resulting NaOH concentration was 4% (*w*/*v*), corresponding to a liquid-to-fiber ratio of 40 mL g^−1^. The two peroxide additions represented 10 and 20% (*v*/*v*) of the stock solution in the final bath and are designated BPJ-3% and BPJ-6%, respectively, following the original experimental nomenclature. These labels should not be interpreted as independently verified final concentrations of pure H_2_O_2_.

Both alkaline peroxide treatments were performed at 70 °C for 1 h with magnetic stirring at 350 rpm. After treatment, the fibers were washed 5–6 times with water until neutral pH and dried at 60 °C for 5 h. No subsequent conditioning at controlled temperature and relative humidity was applied, and residual moisture was not independently measured before the physicochemical analyses. The processing sequence and intended experimental contrasts are summarized in Table 1.

The SPJ-84/SPJ-104 comparison represents the prescribed drying-temperature contrast. BPJ-3% and BPJ-6% compare two peroxide-stock additions with the same stated NaOH concentration, temperature, duration, washing, and final-drying conditions. Comparisons between SPJ-104 and the BPJ specimens necessarily include the complete wet-treatment, washing, and redrying sequence; no alkali-only, peroxide-only, or matched wash/redry controls were available. Because hydrogen peroxide was introduced within an alkaline treatment medium and the BPJ specimens were additionally subjected to renewed wetting, heating, washing to neutral pH, and redrying, several chemical and processing variables changed simultaneously relative to SPJ-104. The observed differences are therefore interpreted as the integrated physicochemical response to the complete alkaline-oxidative processing sequence rather than as a peroxide-specific effect.

### 2.3. Sampling Hierarchy and Experimental Unit

The five-condition physicochemical series was derived from a single artisan-processed batch obtained from the Centro Artesanal de Procesamiento de Paja Toquilla “Barcelona”, Santa Elena, Ecuador. The numbers of individual plants and parent fiber bundles contributing to the artisan batch were not recorded. One condition-level specimen was available for each of PJ-0, SPJ-84, SPJ-104, BPJ-3%, and BPJ-6%. SPJ-84 and SPJ-104 each corresponded to one laboratory-processed fiber portion; the conditioning and drying preparations were not independently repeated. For the two BPJ states, one treatment bath was prepared for each nominal formulation (BPJ-3% and BPJ-6%), with no independently prepared replicate baths. No independently treated replicate specimens were available for any condition. The independent sample size at the specimen or treatment level was therefore n=1 per condition for the main physicochemical series.

For each condition, one parent color ROI and one SEM field were analyzed, while XRD, ATR-FTIR, and TGA were represented by individual comparative profiles. Micro-ROIs, overlapping image windows, regression fits, threshold perturbations, spatial bootstrap resamples, and repeated instrumental observations characterize variation within specimens or images, or in model fitting, and were not treated as independent experimental replicates. The three independent extractions used for the operational fractionation experiment were part of a separate bulk fractionation screen and do not constitute replication of the five-condition physicochemical series.

### 2.4. Relative Image-Based Color Analysis

Relative color appearance was evaluated using the PT Colorimetry Lab workflow, developed in-house by Sebastian Ojeda (Ambato, Ecuador), on photographs acquired under the same reported imaging configuration. RGB channel gains were balanced using a bright, approximately neutral peripheral region as a reference. A certified color target, an instrument-traceable reflectance reference, and complete camera and illumination metadata were not available. Consequently, the resulting $L^{*}$, $a^{*}$, $b^{*}$, and $\Delta E_{00}$ values are interpreted as relative image-derived descriptors within the analyzed image set, not as instrumentally calibrated reflectance color measurements, standardized whiteness values, or color-acceptance criteria [18].

For each condition, a circular parent region of interest (ROI) with a radius of 200 pixels was positioned over a visually homogeneous area. Spatial variability within the parent ROI was sampled using 500 pseudo-random micro-ROIs with a radius of 4 pixels using a pseudo-random number generator with a fixed seed (20260911) to ensure reproducible micro-ROI sampling. sRGB values were linearized, converted to CIE XYZ using a D65 computational reference, and then transformed to CIE 1976 $L^{*}a^{*}b^{*}$ coordinates. Lightness $L^{*}$, green–red coordinate $a^{*}$, blue–yellow coordinate $b^{*}$, chroma $C_{ab}^{*}=\sqrt{a^{*2}+b^{*2}}$, and hue angle were retained as image-derived descriptors. Directional changes in $L^{*}$, $a^{*}$, and $b^{*}$ were calculated relative to PJ-0, and CIEDE2000 differences ($\Delta E_{00}$) were calculated from these image-derived coordinates both relative to PJ-0 and pairwise among the measured conditions [19]. Accordingly, $\Delta E_{00}$ is used here as a relative distance within the image-derived CIELAB representation and not as a standardized perceptual color difference obtained from calibrated reflectance measurements.

The analysis examined the appearance of the parent images, coordinate distributions, the $a^{*}$–$b^{*}$ plane with 95% spatial data ellipses, directional changes, a pairwise $\Delta E_{00}$ matrix, and hue–chroma organization. Micro-ROI dispersion describes within-image heterogeneity and was not treated as independent experimental replication. The complete relative image-based color analysis is presented in the Results and Discussion section.

### 2.5. X-Ray Diffraction

X-ray diffraction measurements were performed using a Rigaku MiniFlex 600 diffractometer (Rigaku Corporation, Akishima, Tokyo, Japan) equipped with a D/teX Ultra2 detector. Patterns were collected over 2θ=5–50° with a sampling interval of 0.01° using Cu Kα radiation (λ=1.5406 Å).

Diffraction profiles were processed in Profex (Nicola Döbelin, Solothurn, Switzerland) [20]. The background was estimated using the statistics-sensitive nonlinear iterative peak-clipping (SNIP) algorithm with a decreasing clipping window and 120 iterations. The estimated background was subtracted from each original diffraction profile to obtain a baseline-corrected pattern. The corrected profiles were subsequently smoothed using a simple moving average (SMA) with a window size of 25 data points, corresponding to a 0.25° interval in 2θ at the experimental sampling step.

The Profex instrument configuration Rigaku-Miniflex-600-DTEXultra2-fds with characteristic Cu radiation was used to describe the Rigaku MiniFlex 600/D/teX Ultra2 measurement geometry during profile processing. However, no separate crystalline line-broadening reference standard was measured, and no explicit instrumental-broadening correction was applied to the QualX-derived FWHM values used in the Scherrer calculation.

Peak positions and full widths at half maximum (FWHM) were obtained using the peak-search routine implemented in QualX (Institute of Crystallography, CNR, Bari, Italy) [21]. The FWHM used in the Scherrer calculation was that of the principal cellulose-associated diffraction feature near 2θ≈22°. It therefore represents the observed peak width after the stated baseline subtraction and smoothing, rather than an independently instrument-corrected line width. No separate multi-peak deconvolution or Rietveld phase refinement was used to derive the reported Scherrer dimensions. Accordingly, the resulting $D_{\mathrm{app}}$ values are treated only as apparent coherent-domain descriptors for comparison, not as absolute, instrument-corrected crystallite sizes.

Interplanar spacing was calculated using Bragg’s law,

(1) $$d=\frac{\lambda }{2\sin\theta }.$$

An apparent coherent-domain dimension was estimated using the Scherrer equation [22],

(2) $$D_{\mathrm{app}}=\frac{K\lambda }{\beta \cos\theta },$$

where K=0.94 is the Scherrer shape factor, λ is the Cu Kα wavelength, β is the FWHM of the principal cellulose-associated diffraction peak expressed in radians, and θ is the corresponding Bragg angle.

The apparent Segal crystallinity index was calculated as follows [23]:

(3) $${\mathrm{CrI}}_{\mathrm{app}}=100\frac{I_{200}-I_{\mathrm{am}}}{I_{200}},$$

where $I_{200}$ is the intensity of the principal cellulose-associated diffraction maximum and $I_{\mathrm{am}}$ is the intensity associated with the amorphous contribution used in the Segal construction. Because the Segal method is sensitive to background treatment and peak definition [24,25], ${\mathrm{CrI}}_{\mathrm{app}}$ is interpreted as an empirical comparative index rather than a quantitative crystalline phase fraction. Similarly, $D_{\mathrm{app}}$ is retained as an apparent coherent-domain descriptor for comparison among the measured profiles.

### 2.6. ATR-FTIR Spectroscopy

ATR-FTIR spectra were collected using a PerkinElmer Spectrum Two FT-IR spectrometer (PerkinElmer, Shelton, CT, USA) equipped with a PIKE MIRacle UATR accessory (PIKE Technologies, Madison, WI, USA). Three scans were recorded over 4000–650 cm^−1^. Transmittance data were converted to absorbance using $A=-\log_{10}\left(T/100\right)$ and baseline-corrected using the original processing workflow.

Selected spectral contributions were normalized to the polysaccharide-dominated region near 1030 cm^−1^. Interpretation focused on the broad O–H stretching envelope, aliphatic C–H stretching, the carbonyl-sensitive region near 1730 cm^−1^, the water/conjugated region near 1620 cm^−1^, and overlapping carbohydrate- and lignin-associated bands within the fingerprint region [1,26]. The resulting absorbance ratios are used as comparative spectral descriptors and are not interpreted as direct polymer-composition measurements.

### 2.7. SEM Acquisition and Multiscale Image Analysis

#### 2.7.1. Image Acquisition, Segmentation, and Boundary Representation

Backscattered-electron SEM images were acquired using a Phenom ProX scanning electron microscope (Thermo Fisher Scientific, Eindhoven, The Netherlands) under the same nominal conditions for all five specimen states (PJ-0, SPJ-84, SPJ-104, BPJ-3%, and BPJ-6%). Images were recorded in BSD Full mode at 2000× magnification, using an accelerating voltage of 10 kV, a chamber pressure of 60 Pa, a field width of 259 μm, and working distances close to 8 mm. One SEM field was analyzed for each condition using the in-house SEM Fractal & Texture Analyzer v6.7 workflow, developed by Sebastian Ojeda (Ambato, Ecuador). Each micrograph contained an embedded 50 μm scale bar, which was used for spatial calibration before image analysis. Maintaining the same nominal acquisition settings across the five fields provided a consistent basis for the subsequent scale-dependent image comparisons.

The microscope footer was excluded from the analysis. Intensity normalization used the 0.5th and 99.5th percentiles as robust limits. Phase segmentation was performed using Otsu thresholding, with Li thresholding used as a fallback when the Otsu threshold approached an extreme of the occupied intensity range. Bright- and dark-foreground alternatives were evaluated automatically, and the solution satisfying the workflow’s occupancy and edge-representation criteria was retained. Small isolated objects and holes were removed before extraction of a 3×3 morphological-gradient edge network. The segmented foreground was defined exclusively from image intensity and was not assigned to a specific chemical phase. Likewise, bright features in the BSE images were not attributed to individual elements because no elemental microanalysis was performed.

#### 2.7.2. Boundary and Grayscale Fractal Dimensions

Boundary fractal dimension $D_{B}$ was estimated from the segmented two-dimensional SEM edge network using translated-grid box counting. Box-counting is an established image-based approach for expressing geometrical complexity as a quantitative scale-dependent descriptor and has been applied to SEM-derived microstructural features [27]. For each box size ε, the number of boxes N(ε) intersecting the segmented boundary was counted and averaged over 16 translated grid origins. Twenty-four target scales were distributed logarithmically between 2 pixels and one quarter of the minimum image dimension, after removing duplicate integer scales. Contiguous scaling windows containing at least five scales were evaluated for linearity, scale span, and local-slope stability.

The boundary fractal dimension was estimated from the slope of the scaling relation

(4) $$D_{B}=\frac{d\ln N(\varepsilon )}{d\ln(1/\varepsilon )},$$

where N(ε) is the number of boxes of characteristic size ε required to intersect the segmented boundary. In practice, $D_{B}$ was obtained by linear regression of lnN(ε) against ln(1/ε) over the selected scaling window. Regression standard errors and confidence intervals associated with the box-counting fit describe uncertainty in the fitted scaling relation for the individual image only. They do not quantify between-field, between-specimen, or treatment-level uncertainty and were not used for treatment-level inference.

Grayscale fractal complexity was evaluated independently using differential box counting following Sarkar and Chaudhuri [28]. The resulting $D_{\mathrm{DBC}}$ describes multiscale variation of the grayscale-intensity surface, whereas $D_{B}$ describes the geometry of the threshold-defined edge network. These descriptors capture different image properties and are not direct measurements of three-dimensional surface roughness. Local $D_{\mathrm{DBC}}$ values were also calculated in overlapping windows to characterize spatial variation within each field.

#### 2.7.3. Lacunarity and Multifractal Organization

Gliding-box lacunarity was evaluated over 18 logarithmically distributed box sizes [29],

(5) $$\Lambda \left(r\right)=\frac{\mathrm{Var}[M(r)]}{E{\left[M\left(r\right)\right]}^{2}}+1,$$

where M(r) is the occupied mass within a box of size *r*. Because lacunarity depends on phase occupancy and framing, it is interpreted as a descriptor of gap heterogeneity within the segmented two-dimensional field rather than as volumetric porosity.

Multifractal properties of the positive grayscale-intensity measure were calculated using the direct Chhabra–Jensen formalism [30]. The largest centered square with a power-of-two side length was analyzed without interpolation. Exact dyadic box sizes and 41 moment orders spanning q=−5 to 5 were used to estimate generalized dimensions $D_{q}$, singularity strengths α(q), and the singularity spectrum f(α). Results were retained when the capacity dimension was consistent with two-dimensional support, $D_{q}$ was approximately non-increasing with *q*, at least four dyadic scales were available, and median regression $R^{2}$ values for the partition and singularity relations were at least 0.95. Information dimension $D_{1}$, correlation dimension $D_{2}$, and spectrum width Δα were used for comparative interpretation.

#### 2.7.4. Orientation, Spectral Texture, and Intensity Entropy

Directional organization was estimated from the grayscale gradient field after Gaussian smoothing (σ=1 pixel) and Sobel differentiation. Structural direction $\theta_{i}$ was defined perpendicular to the local gradient and weighted by gradient magnitude $w_{i}$. Axial anisotropy was calculated as

(6) $$A=\frac{\left|\sum_{i}w_{i}\exp\left(2i\theta_{i}\right)\right|}{\sum_{i}w_{i}}.$$

Orientation distributions were summarized over 0–180°. Because fiber bundles were not registered to a common longitudinal axis before imaging, absolute peak angles were treated as image-coordinate descriptors; the rotation-invariant magnitude *A* was used for comparisons between conditions.

The radially averaged two-dimensional power spectral density (PSD) was used to characterize frequency-domain texture. After mean subtraction and application of a two-dimensional Hann window, Fourier power was binned radially on a logarithmic frequency axis and fitted to $P\left(k\right)\propto k^{-\beta }$ over 0.02–0.35 cycles pixel^−1^. Shannon entropy was calculated from the 8-bit intensity histogram as $H=-\sum_{i}p_{i}\log_{2}p_{i}$ [31]. Rotation-averaged gray-level co-occurrence contrast and homogeneity values were retained as supporting descriptive measures.

Scale-dependent comparisons were restricted to the five fields acquired under the same nominal magnification and field-width conditions. The scale range for each descriptor is reported in the corresponding subsection.

The SEM-derived quantities were treated as complementary descriptors of the same measured field, not as independent experimental outcomes. For interpretation, the descriptors were grouped conceptually into boundary/occupancy measures ($D_{B}$, foreground occupancy, and lacunarity), grayscale multiscale measures ($D_{\mathrm{DBC}}$, multifractal descriptors, PSD, and entropy), and directional measures (orientation distributions and axial anisotropy). Agreement among descriptors was not used to construct a composite score, conduct a statistical vote, or increase the effective sample size. Because all descriptors for a condition originate from the same SEM field, agreement among them does not constitute independent replication.

Foreground occupancy was calculated as the fraction of analyzed image pixels assigned to the segmented foreground. Across the five selected SEM fields, foreground occupancy ranged from 12.3% to 42.7%. Because boundary-based descriptors depend not only on boundary geometry but also on the amount, distribution, and framing of segmented foreground within the image, occupancy was retained as a contextual descriptor when interpreting $D_{B}$ and lacunarity.

#### 2.7.5. Sensitivity and Within-Image Resampling

Segmentation sensitivity was evaluated by recomputing $D_{B}$ at threshold offsets from −20 to +20 gray levels in steps of 5. Within-image heterogeneity was also summarized using two hundred spatial bootstrap resamples. The threshold-perturbation analysis evaluates within-image sensitivity to plausible segmentation choices and does not assess sensitivity to independent field selection or specimen-to-specimen variation. Because only one SEM field was available per condition, the effects of alternative field framing and between-field heterogeneity could not be quantified independently. These operations were not treated as independent experimental replicates.

### 2.8. Thermogravimetric Analysis

Thermogravimetric analysis was performed using a TA Instruments TGA55 (TA Instruments, New Castle, DE, USA) with 100 μL platinum pans under nitrogen. Samples were heated to approximately 800 °C at 10 °C min^−1^. Initial masses were 2.043, 2.109, 1.847, 1.400, and 2.996 mg for PJ-0, SPJ-84, SPJ-104, BPJ-3%, and BPJ-6%, respectively. Comparisons were based on normalized residual mass *W*, with DTG expressed as dW/dT.

The principal degradation-rate temperature, $T_{\max}$, was defined as the temperature of the main DTG minimum after excluding the initial stabilization region. Mass loss was also evaluated over common intervals of 30–150, 150–250, 250–380, 380–600, and 600–793 °C. The remaining mass near 793 °C is referred to as pyrolytic residue because measurements under nitrogen do not distinguish carbonaceous from inorganic contributions. These intervals represent overlapping degradation domains and are not assigned quantitatively to cellulose, hemicellulose, or lignin [32].

### 2.9. Operational Bulk Fraction Recovery

A separate bulk fractionation screen was performed in three independent experiments. Dried paja toquilla was ground in a MODEL-150 multifunctional crusher at 22,000 rpm using three 5-min cycles separated by 10-min cooling intervals and passed through a 100 μm sieve. The ground material was extracted with 4% NaOH at 70 °C for 1 h under gentle agitation. The alkali-insoluble residue was filtered, washed to approximately neutral pH, and designated the cellulose-enriched fraction.

The alkaline filtrate was acidified with HCl to approximately pH 2, allowed to stand for 90 min, and centrifuged. The resulting precipitate was dried at 60 °C for 8 h and designated the lignin-enriched fraction. Absolute ethanol was then added to the supernatant remaining after lignin precipitation at a supernatant:ethanol volume ratio of 1:3 to precipitate a hemicellulose-enriched fraction. The fraction names therefore denote operationally enriched materials rather than chemically pure cellulose, hemicellulose, or lignin.

Recovery was calculated as

(7) $$Y_{i}=100\frac{m_{i}}{m_{0}},$$

where $m_{i}$ is the recorded mass of the recovered operational fraction and $m_{0}$ is the initial dry mass of the starting material. The acid-precipitated lignin-enriched fraction was dried at 60 °C for 8 h; however, a verified constant-mass endpoint was not established for all recovered fractions. No component-specific purity correction, ash correction, or residual-salt correction was applied.

Consequently, the recovered masses should not be interpreted as a closed analytical dry-mass balance. Residual moisture, retained soluble or inorganic species, residual salts, and co-precipitation or co-recovery of chemically different constituents may contribute to apparent total recoveries above 100%. Normalized shares were calculated relative to the total recovered material in each experiment and are used only as descriptors of the operational fractionation screen. This procedure should not be equated with validated structural-carbohydrate and lignin assays based on hydrolysis and component-specific quantification [33]; the fractionation experiment was not performed separately for each processing condition.

### 2.10. Statistical Scope and Interpretation

Bulk fraction recovery is reported as mean ± standard deviation from three independent extractions performed specifically for the operational fractionation experiment. In contrast, the principal five-condition physicochemical series contained one independently prepared specimen per condition (n=1). Consequently, between-specimen or between-treatment variance could not be estimated, and no treatment-level confidence intervals, hypothesis tests, or claims of statistical significance are reported.

Color micro-ROI distributions, regression errors, threshold perturbations, overlapping windows, and spatial bootstrap samples quantify within-image, within-specimen, or model-fitting variability only. They do not increase the number of independent experimental units. Accordingly, direct comparisons between BPJ-3% and BPJ-6% describe the two measured condition-level specimens and do not estimate between-treatment variance or establish reproducible superiority of either nominal formulation.

In the five-condition physicochemical series, each condition was represented by one measured XRD profile and one TGA/DTG profile. Independent replicate profiles and experimental repeatability estimates were not available for these measurements. Consequently, differences among apparent diffraction and thermal descriptors are reported as specimen-level numerical observations and are neither accompanied by treatment-level variance estimates nor interpreted as reproducible processing trends. Cross-technique agreement is therefore interpreted as complementary specimen-level evidence rather than as a substitute for independent replication.

## 3. Results and Discussion

### 3.1. Material Context and Operational Lignocellulosic Recovery

PJ-0 is an artisan-processed reference rather than pristine plant tissue. Manual subdivision, hydrothermal cooking, drainage, and drying precede the laboratory treatments and define the initial physicochemical state of the experimental series. Accordingly, SPJ-84 and SPJ-104 represent the prescribed drying-temperature contrast, whereas BPJ-3% and BPJ-6% compare two peroxide-stock additions within the same alkaline treatment sequence. Because hydrogen peroxide was introduced into an alkaline medium and processing of SPJ-104 into the BPJ states also involved renewed wetting, heating, washing to neutral pH, and redrying, the SPJ-104-to-BPJ comparison is interpreted as the integrated physicochemical response to the complete alkaline-oxidative processing sequence rather than as a peroxide-specific effect.

**Figure 2:**
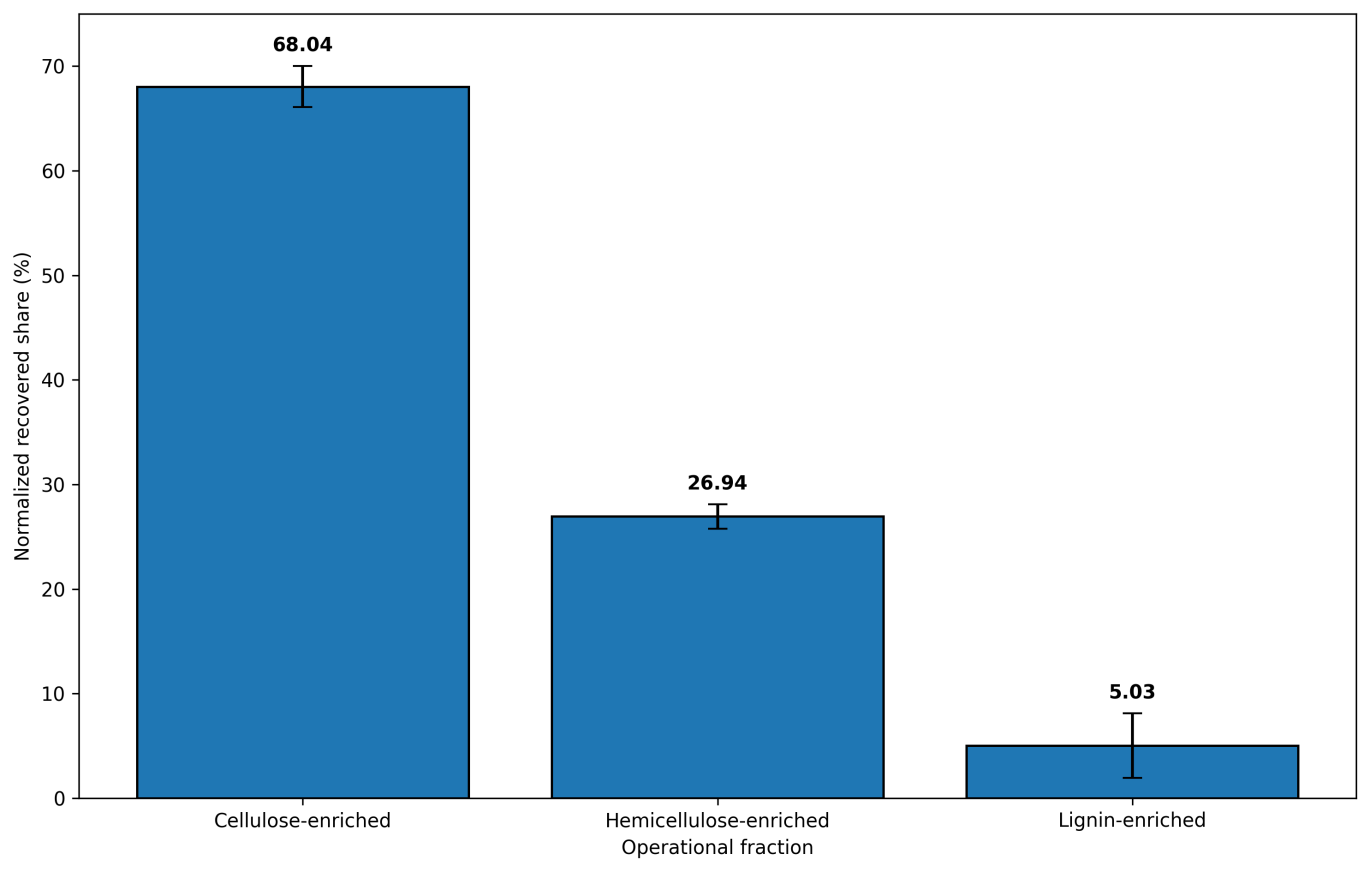
Normalized shares of the operationally recovered cellulose-enriched, hemicellulose-enriched, and lignin-enriched fractions. Values are shown as mean ± standard deviation across the three independent fractionation experiments. The fractions represent operationally enriched materials rather than chemically pure constituents.

The independent fraction-recovery screen was dominated by carbohydrate-enriched material (Table 2; Figure 2). For an initial dry mass of 1.00 g, the mean recovered masses were 0.6790 ± 0.0517 g for the cellulose-enriched fraction, 0.2686 ± 0.0172 g for the hemicellulose-enriched fraction, and 0.0524 ± 0.0354 g for the acid-precipitated lignin-enriched fraction. After normalization to total recovered material, these fractions accounted for approximately 68, 27, and 5%, respectively. These results provide an operational fractionation context for the polysaccharide-dominated FTIR response and cellulose-associated diffraction features discussed below, but do not constitute a validated compositional analysis.

Across the three independent operational fractionation experiments, total recovery ranged from 88.99 to 109.68%. Total recovery above 100% indicates that the procedure did not yield a closed analytical mass balance. The available summary dataset contains the aggregate fraction statistics and the observed total-recovery range, but not a validated replicate-level table of the individual recovered fraction masses; no individual fraction values were reconstructed retrospectively.

Several factors, which are not mutually exclusive, may contribute to the observed mass-balance non-closure. A verified constant-mass drying endpoint was not documented for all recovered fractions, and residual moisture may therefore contribute to the recorded mass. In addition, the operational precipitation and separation steps are not component-specific and may result in co-precipitation or co-recovery of multiple constituents. Retained soluble or inorganic species, including residual salts, may also contribute because no ash, component-purity, or residual-salt correction was performed. The reported recoveries and normalized shares are therefore interpreted as operational fraction-recovery descriptors rather than quantitative cellulose, hemicellulose, or lignin contents. Because the fractionation experiment was not performed separately for each processing condition, it cannot quantify treatment-specific cellulose enrichment or lignin removal. Its role in the present study is therefore contextual rather than mechanistic.

### 3.2. Relative Image-Based Color Response

#### 3.2.1. Visible Appearance and CIELAB Coordinates

**Figure 3:**
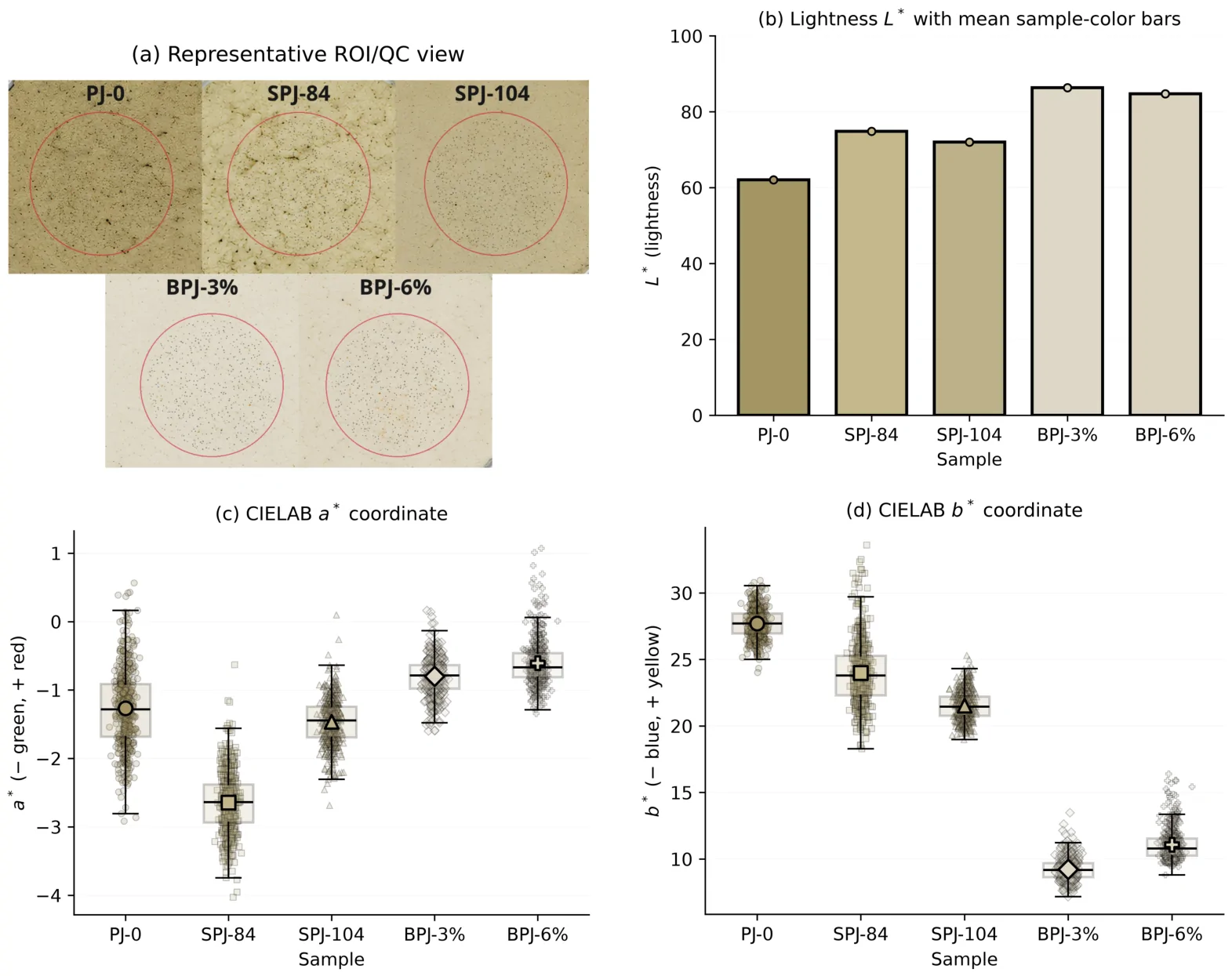
Relative image-based color descriptors: (**a**) representative ROIs; (**b**) image-derived lightness $L^{*}$; (**c**) CIELAB coordinate $a^{*}$ (negative values indicate green and positive values indicate red); and (**d**) blue–yellow coordinate $b^{*}$. Red circular outlines in panel (**a**) delimit the parent ROIs. In panel (**b**), bar heights and circular markers indicate mean $L^{*}$ values, and bar fills represent the mean image-derived sample colors. In panels (**c**,**d**), circles, squares, triangles, diamonds, and crosses denote PJ-0, SPJ-84, SPJ-104, BPJ-3%, and BPJ-6%, respectively. Small markers represent individual micro-ROI values, whereas large markers indicate means. Boxes span the 25th–75th percentiles, central lines indicate medians, and whiskers extend to the most extreme values within 1.5 times the interquartile range of the box limits. These distributions summarize within-image micro-ROI variability and do not represent between-specimen or treatment-level uncertainty.

The parent ROIs and corresponding image-derived CIELAB distributions show differences in relative lightness and chromatic coordinates among the measured specimen images (Figure 3). PJ-0 exhibited the darkest cream-yellow appearance in the analyzed image set, the dried specimens showed higher image-derived lightness, and the BPJ specimens occupied a paler, lower-chroma region. The values in Table 3 are therefore interpreted as relative optical descriptors of the selected images rather than as instrumentally calibrated color measurements or treatment means from independent replicates.

Image-derived lightness was approximately 62 in PJ-0, 75 in SPJ-84, and 72 in SPJ-104, so the two dried specimen images did not follow the same numerical ordering as increasing drying temperature. The BPJ images showed a larger relative optical displacement with respect to SPJ-104. The BPJ-3% image had $L^{*}\approx 86$, whereas the BPJ-6% image had $L^{*}\approx 84$. These numerical differences refer to the individual analyzed images and are not interpreted as evidence of a reproducible difference between the nominal formulations. Because peroxide exposure occurred within an alkaline medium and was accompanied by renewed wetting, heating, washing to neutral pH, and redrying, the optical contrast between SPJ-104 and the BPJ states is interpreted as the integrated response to the complete alkaline-oxidative processing sequence rather than as a peroxide-specific effect.

All $a^{*}$ values remained negative, with SPJ-84 showing the largest greenward displacement ($a^{*}=-2.65$). The BPJ images had $a^{*}$ values closer to zero (−0.80 for BPJ-3% and −0.61 for BPJ-6%), whereas the numerical differences in $b^{*}$ were more pronounced. The image-derived yellow coordinate decreased from 27.71 in PJ-0 to 23.98 and 21.54 in SPJ-84 and SPJ-104, respectively, and reached 9.23 and 11.08 in BPJ-3% and BPJ-6%. Numerically, the BPJ-3% image therefore showed $L^{*}\approx 86$ and $b^{*}=9.23$, whereas the BPJ-6% image showed $L^{*}\approx 84$ and $b^{*}=11.08$. These differences were obtained from one image per condition and should be considered alongside the small pairwise separation between the two BPJ specimens in the image-derived color space.

#### 3.2.2. Color Difference and Directional Changes

**Figure 4:**
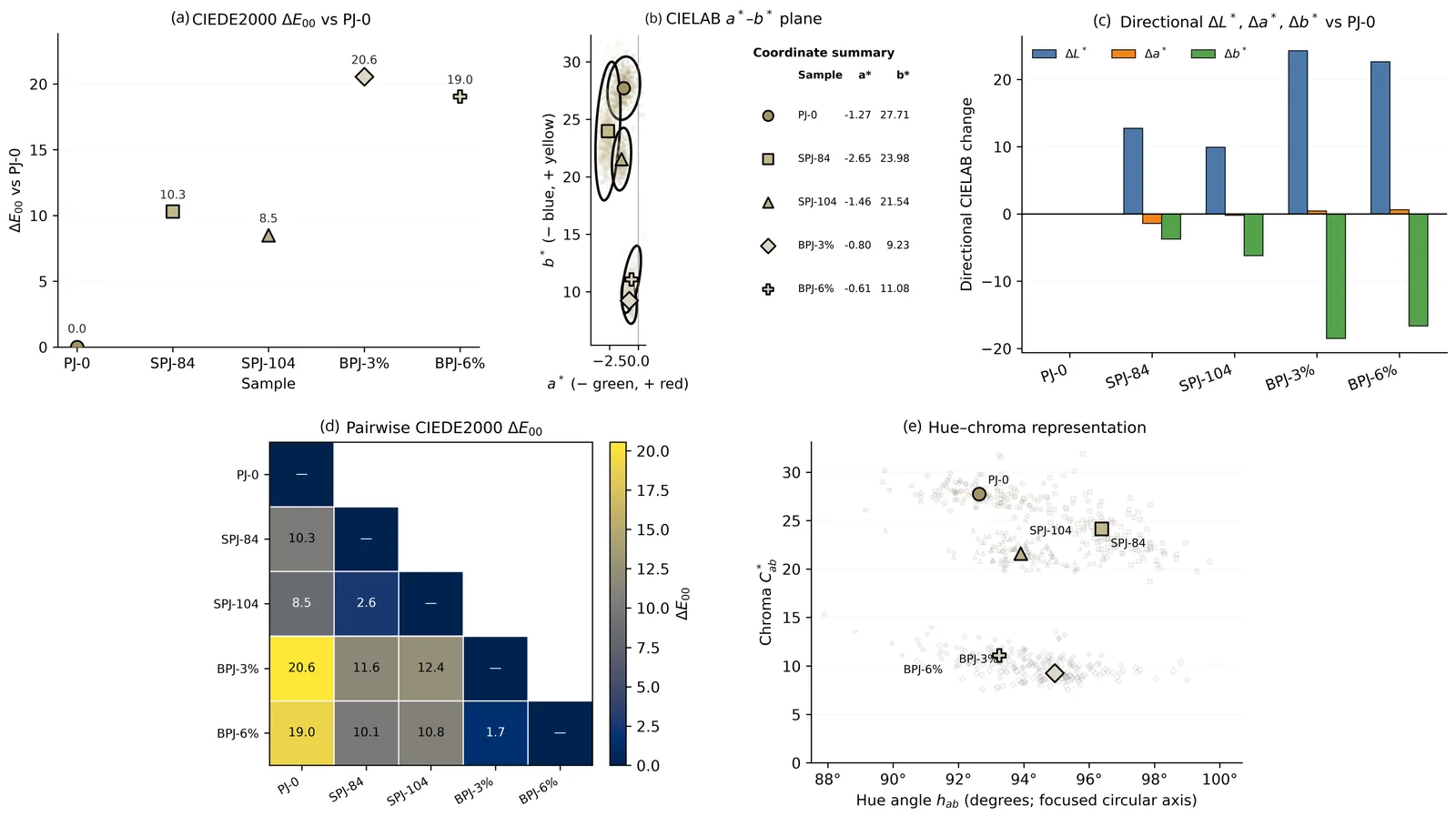
Complementary relative image-based color response: (**a**) $\Delta E_{00}$ relative to PJ-0; (**b**) $a^{*}$–$b^{*}$ distributions with 95% ellipses; (**c**) directional color changes relative to PJ-0; (**d**) pairwise $\Delta E_{00}$; and (**e**) hue–chroma organization. In panels (**a**,**b**,**e**), circles, squares, triangles, diamonds, and crosses denote PJ-0, SPJ-84, SPJ-104, BPJ-3%, and BPJ-6%, respectively. Small translucent markers in panels (**b**,**e**) represent individual micro-ROI values; large markers indicate specimen-level summaries. Black ellipses in panel (**b**) summarize the 95% spatial distributions of micro-ROIs. In panel (**c**), blue, orange, and green bars represent $\Delta L^{*}$, $\Delta a^{*}$, and $\Delta b^{*}$, respectively. Panel (**d**) shows unique pairwise comparisons in the lower triangle; diagonal dashes denote self-comparisons, for which $\Delta E_{00}=0$. Micro-ROI distributions and ellipses describe within-image heterogeneity and do not represent independent specimen replication or treatment-level confidence regions.

The CIEDE2000 calculation relative to PJ-0 combines the image-derived coordinate differences into a single relative color-distance descriptor within the analyzed image set (Figure 4a). SPJ-84 and SPJ-104 showed image-derived $\Delta E_{00}$ values of 10.3 and 8.5, whereas BPJ-3% and BPJ-6% showed values of 20.6 and 19.0, respectively. The directional changes in panel (c) indicate that the separation is driven primarily by positive $\Delta L^{*}$ and negative $\Delta b^{*}$, with a comparatively small $\Delta a^{*}$ contribution.

The pairwise matrix (panel d) distinguishes the larger image-space displacement of the BPJ specimens from the comparatively small difference between the two BPJ images. SPJ-84 and SPJ-104 differed by 2.6, whereas BPJ-3% and BPJ-6% differed from their common SPJ-104 precursor by 12.4 and 10.8, respectively. The two BPJ images were separated by only $\Delta E_{00}=1.7$ within the image-derived CIELAB representation, despite the different nominal peroxide-stock additions. These values are used for relative comparison of the analyzed images and not as standardized reflectance color, whiteness, or acceptance measurements.

#### 3.2.3. Within-Image Heterogeneity and Hue–Chroma Organization

The $a^{*}$–$b^{*}$ distributions complement the central color values with information about spatial variability (Figure 4b). SPJ-84 spans a comparatively broad range of local coordinates, SPJ-104 is shifted toward lower yellowness, and both bleached ROIs occupy a clearly separated low-$b^{*}$ region. The displacement therefore reflects the sampled ROI as a whole, rather than a small number of extreme pixels. These distributions describe within-image heterogeneity, however, and should not be interpreted as population-level treatment statistics.

Hue remained confined to a narrow yellow-associated range (approximately 92.6–96.3°), whereas chroma decreased from approximately 27.7 in PJ-0 to 9–11 after bleaching (Figure 4e). The visual transformation was therefore dominated by increased lightness and reduced chromatic intensity rather than by a substantial change in hue angle. Across the coordinate plots, directional changes, spatial distributions, and pairwise matrix, the two BPJ specimen images occupied a similar pale, low-chroma region of the image-derived color space, with only a small pairwise separation ($\Delta E_{00}=1.7$).

Because BPJ-3% and BPJ-6% differed in nominal peroxide-stock addition but were separated by only $\Delta E_{00}=1.7$ in the analyzed image set, the available photographs are interpreted as showing similar measured appearance. In the absence of independently acquired standardized color measurements and between-specimen variability, the present data do not support a conclusion that either nominal BPJ formulation produces superior whitening. Relative image-based color analysis alone cannot distinguish among changes in chromophoric environments, selective removal of colored constituents, surface scattering, and acquisition effects; the diffraction, spectroscopic, morphological, and thermal analyses therefore provide complementary specimen-level descriptors.

### 3.3. Cellulose-Associated Diffraction and Apparent Ordering

**Figure 5:**
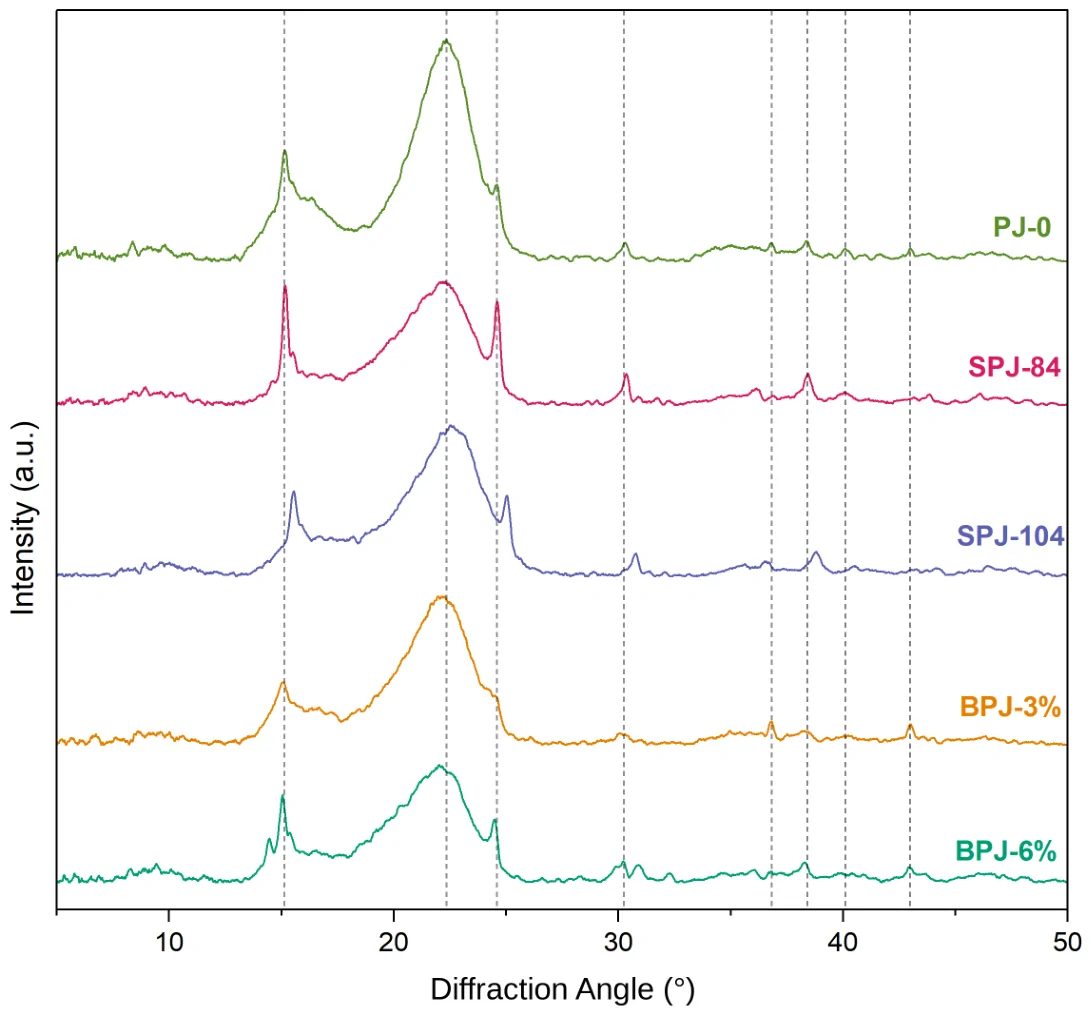
XRD profiles of the five processing conditions, vertically displaced for comparison. Dashed lines indicate the main diffraction regions analyzed.

All five XRD profiles showed the broad semicrystalline signature expected for cellulose-rich lignocellulosic fibers, with a lower-angle contribution near 15–16° and a dominant feature near 22° (Figure 5). The persistence of this pattern is consistent with a cellulose-I-like framework across the processing series. However, the heterogeneous nature of the fibers, preferred orientation, and overlap among cellulose and amorphous contributions preclude an absolute phase assignment. In particular, the absence of a prominent feature near 12° argues against a large-scale cellulose-I-to-cellulose-II conversion but does not exclude minor contributions [25].

PJ-0 exhibited the narrowest FWHM and the highest ${\mathrm{CrI}}_{\mathrm{app}}$ of the measured profiles (Table 4). Within the controlled-drying pair, SPJ-104 showed a slightly narrower principal feature, a larger $D_{\mathrm{app}}$, and a higher ${\mathrm{CrI}}_{\mathrm{app}}$ than SPJ-84. These are specimen-level differences among individual processed diffraction profiles; the accompanying peak-position shift should be interpreted cautiously because packing, preferred orientation, overlapping contributions, and specimen geometry can all influence the fitted maximum.

Across the complete series of measured XRD profiles, $D_{\mathrm{app}}$ ranged from approximately 2.43 to 2.71 nm and ${\mathrm{CrI}}_{\mathrm{app}}$ from 74.5 to 85.8%; within the BPJ pair, BPJ-3% showed $D_{\mathrm{app}}=2.65$ nm and ${\mathrm{CrI}}_{\mathrm{app}}=81.2\%$, whereas BPJ-6% showed $D_{\mathrm{app}}=2.48$ nm and ${\mathrm{CrI}}_{\mathrm{app}}=78.9\%$. Because only one XRD profile was available per condition, with no independent estimate of repeatability, these numerical differences are interpreted as characteristics of the measured profiles rather than evidence of a reproducible processing-induced change in cellulose ordering. Preferential modification of disordered or non-cellulosic domains is one possible context for changes in relative diffraction intensity, but the present data do not demonstrate formation of new crystalline cellulose or quantify lignin removal.

The Segal index is particularly sensitive to the analytical procedure. As demonstrated by Park et al. [24], cellulose crystallinity values can vary substantially with baseline treatment, peak definition, and the method used to separate crystalline and non-crystalline diffraction contributions. The present ${\mathrm{CrI}}_{\mathrm{app}}$ values are therefore used only as internally consistent apparent indices for the measured profiles and should not be interpreted as quantitative crystalline mass fractions or compared directly with crystallinity percentages obtained using different analytical procedures.

### 3.4. ATR-FTIR: Modification Within a Retained Polysaccharide Fingerprint

**Figure 6:**
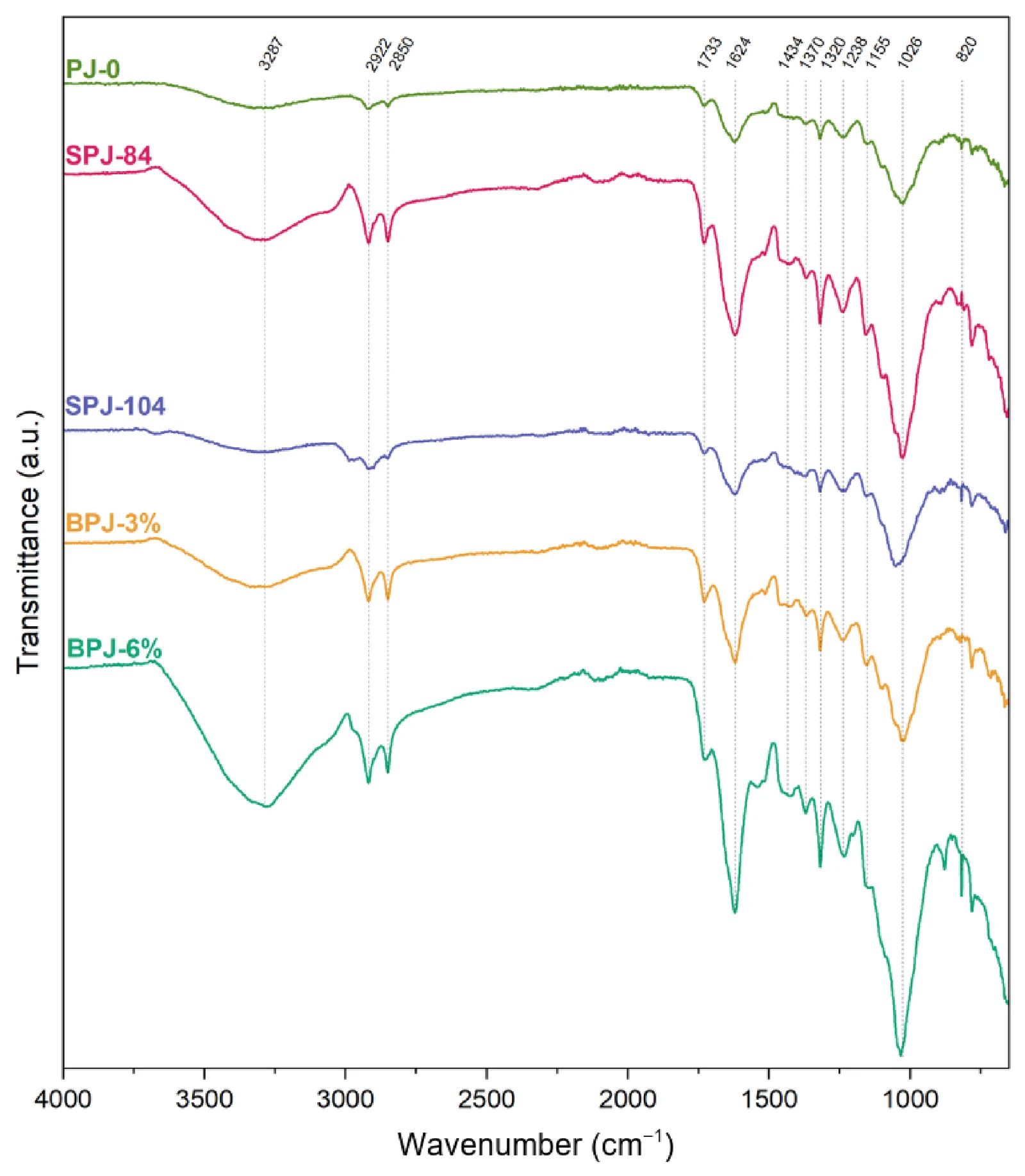
ATR-FTIR spectra of the five processing conditions, vertically displaced for comparison.

The ATR-FTIR spectra retained the broad O–H envelope, aliphatic C–H contributions, and the dominant carbohydrate-associated fingerprint region across all five conditions (Figure 6). Features near 1155 and 1026 cm^−1^ remained prominent after bleaching, supporting the XRD evidence that a polysaccharide-rich framework persisted throughout processing. Because lignocellulosic spectra contain extensive band overlap, however, individual absorptions cannot be assigned uniquely to a single polymer component [26].

The measured ATR-FTIR profiles differed in the O–H- and water-sensitive spectral regions. From SPJ-84 to SPJ-104, both $A_{\mathrm{OH}}/A_{1030}$ and $A_{1620}/A_{1030}$ decreased (Table 5). These changes may reflect differences in hydrogen-bonding environments, water-sensitive spectral contributions, ATR contact, or a combination of these factors. Because residual moisture was not independently measured and no controlled post-drying equilibration was applied, these ratios are not interpreted as quantitative evidence of lower water content in SPJ-104.

The corresponding ratios were higher in the BPJ specimens than in SPJ-104. Although this difference is compatible with changes arising during renewed wetting, washing, redrying, and modification of surface accessibility, the present measurements cannot establish whether hydration state accounts for the difference. The ratios are therefore used only as relative spectral descriptors for comparison.

The BPJ states differed from SPJ-104 in their relative spectral response. The $A_{1235}/A_{1030}$ ratio was 0.228 in SPJ-104, 0.204 in BPJ-3%, and 0.178 in BPJ-6%, indicating modification of the non-cellulosic/carbohydrate spectral environment across the measured specimen states. The carbonyl-sensitive $A_{1730}/A_{1030}$ ratio differed among the same profiles, with values of 0.196 in SPJ-104, 0.396 in BPJ-3%, and 0.247 in BPJ-6%. Changes in carbonyl-associated bands are chemically compatible with reactions that may occur under alkaline and oxidative conditions. However, because peroxide exposure occurred within an alkaline medium and was accompanied by renewed wetting, heating, washing, and redrying, the measured spectral differences represent the integrated chemical and processing response of the BPJ sequence and do not resolve the specific contribution of peroxide. These ratios therefore describe relative spectral reorganization and do not constitute a selective assay of lignin or hemicellulose removal.

Comparable peroxide-based studies on wheat straw and almond-shell-derived fibers report coordinated chemical and morphological changes during cellulose isolation and bleaching [11,12]. Those systems provide mechanistic context, but their removal efficiencies cannot be transferred to *Carludovica palmata*. The key result here is that BPJ-3% and BPJ-6% developed distinct relative spectral fingerprints while preserving the principal polysaccharide bands, consistent with different degrees or modes of matrix modification rather than wholesale loss of the cellulose-rich framework.

### 3.5. SEM Morphology and the Image-Processing Response

**Figure 7:**
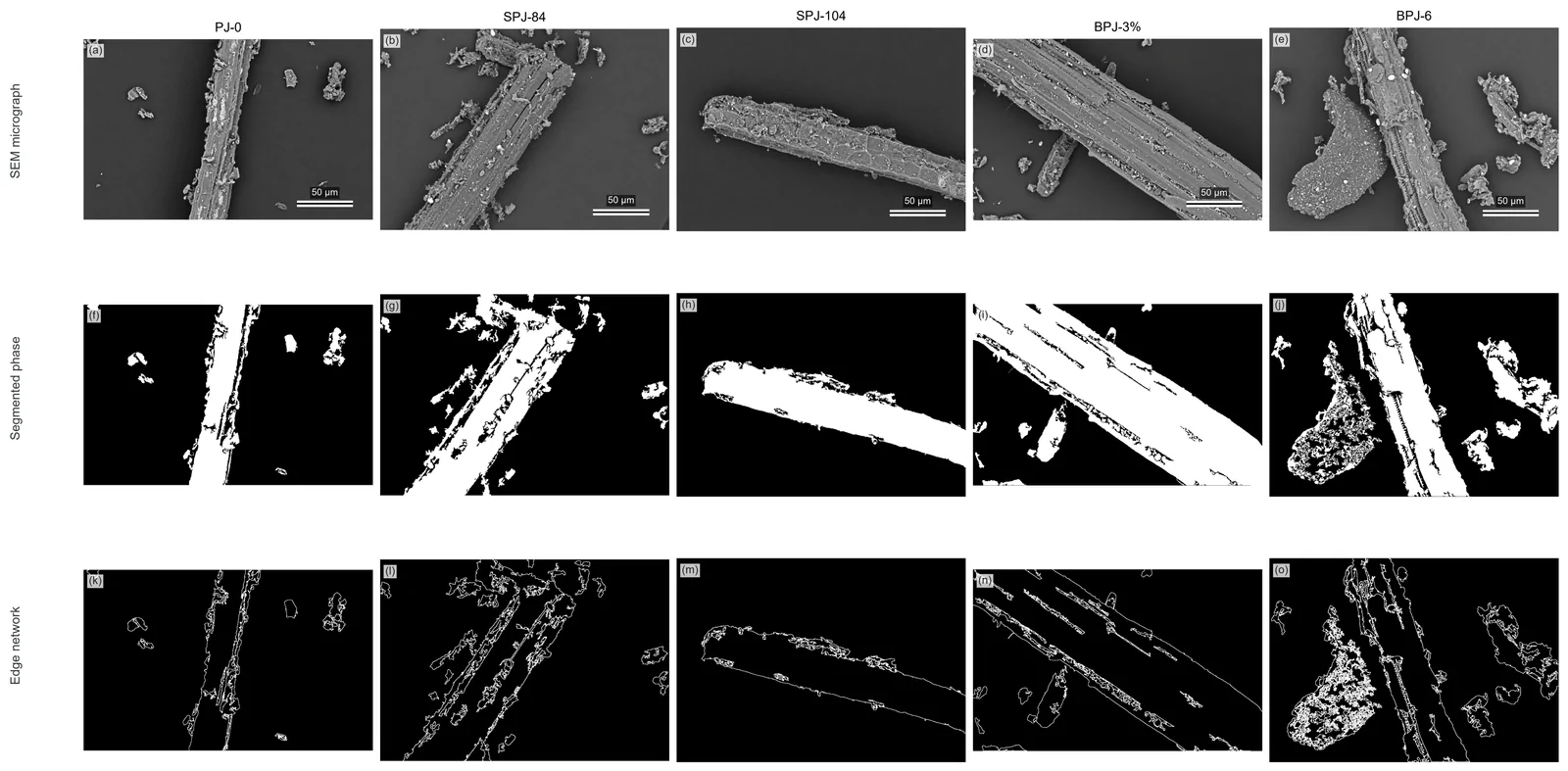
SEM image-processing matrix. (**a**–**e**) BSE micrographs of PJ-0, SPJ-84, SPJ-104, BPJ-3%, and BPJ-6%; (**f**–**j**) segmented foregrounds; and (**k**–**o**) extracted edge networks. Conditions: 2000×, 10 kV, 60 Pa, and 259 μm field width. Scale bars: 50 μm. Each column represents one measured field.

The SEM processing matrix links the visual morphology to the segmented foreground and extracted boundary network (Figure 7). PJ-0 is dominated by a narrow, continuous longitudinal bundle with limited detached material. SPJ-84 retains the main bundle but exhibits greater edge irregularity, local opening, and detached fragments, whereas SPJ-104 appears comparatively compact and continuous with a simpler external contour. The two selected BPJ fields differed visibly: BPJ-3% showed extensive longitudinal exposure with a dominant elongated bundle, whereas BPJ-6% displayed fibrillated edges, detached fragments, and irregular structures distributed across a more heterogeneous field.

The selected fields cannot be adequately described by a simple smooth/rough classification. Their segmented morphologies differ in the distribution of longitudinal structures, disconnected contours, and detached features. The contrast between BPJ-3% and BPJ-6% is therefore treated as a field-level observation: despite their similar measured appearance in the relative image-based color analysis, the two selected fields differed in directional and boundary organization. This optical–morphological contrast provides the rationale for the multiscale image analysis that follows.

Foreground occupancy was 12.3, 21.7, 18.1, 42.7, and 26.3% for PJ-0, SPJ-84, SPJ-104, BPJ-3%, and BPJ-6%, respectively. The high occupancy of BPJ-3% reflects the large fraction of the field occupied by the dominant bundle, whereas the BPJ-6% foreground is distributed among the main bundle and multiple detached structures. Occupancy is therefore a geometric property of the analyzed field rather than a direct damage metric and must be considered jointly with the boundary and texture descriptors.

### 3.6. Fractal and Multifractal Organization of SEM Texture

#### 3.6.1. Boundary Geometry and Grayscale Complexity

**Figure 8:**
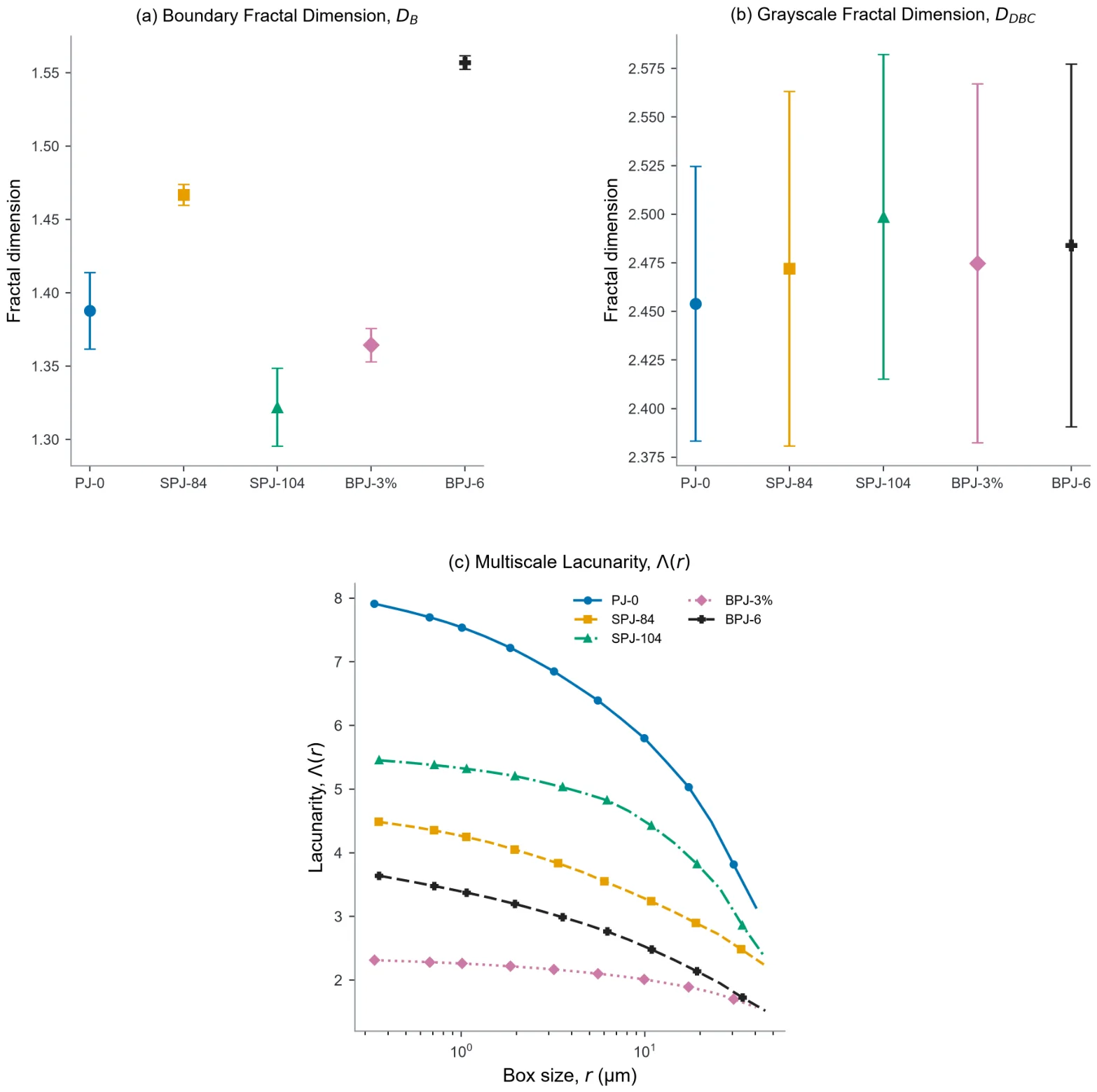
Fractal and spatial-heterogeneity analysis of the five SEM fields: (**a**) boundary fractal dimension $D_{B}$; (**b**) grayscale differential-box-counting dimension $D_{\mathrm{DBC}}$; and (**c**) scale-dependent lacunarity Λ(r). Fitted intervals describe regression uncertainty for the individual image fits only and do not represent between-field, between-specimen, or treatment-level uncertainty.

Among the boundary descriptors, $D_{B}$ showed substantial numerical differences between several of the selected SEM images (Figure 8; Table 6). At the nominal segmentation threshold, $D_{B}$ decreased in the following order: BPJ-6% (1.5570) > SPJ-84 (1.4667) > PJ-0 (1.3877) > BPJ-3% (1.3643) > SPJ-104 (1.3219). However, foreground occupancy differed substantially among the selected fields (12.3–42.7%), and the threshold-perturbation analysis showed that the numerical ordering of $D_{B}$ was not invariant to all plausible segmentation offsets. The boundary-fractal values are therefore interpreted as descriptors of the individual segmented fields rather than as a robust treatment ranking. These values quantify two-dimensional boundary complexity, not three-dimensional surface roughness.

The boundary fits were highly linear over the selected scaling windows ($R^{2}=0.9980$–1.0000), with 23 effective scales spanning approximately 2.08–2.10 decades. This supports the internal consistency of the fitted scaling relation but does not imply invariance to segmentation or field selection; threshold sensitivity is evaluated separately below.

Grayscale complexity followed a different pattern. $D_{\mathrm{DBC}}$ varied only from 2.4540 to 2.4987, with strongly overlapping regression intervals (Figure 8b). SPJ-104 exhibited the highest $D_{\mathrm{DBC}}$ despite having the lowest boundary dimension, highlighting that the two metrics describe different aspects of image structure: $D_{\mathrm{DBC}}$ integrates multiscale variation across the grayscale surface, whereas $D_{B}$ describes the geometry of a threshold-defined edge network. Their divergence is therefore informative and should not be reduced to a single notion of “roughness”.

#### 3.6.2. Scale-Dependent Lacunarity

Mean lacunarity decreased in the order PJ-0 (6.330) > SPJ-104 (4.611) > SPJ-84 (3.615) > BPJ-6% (2.801) > BPJ-3% (2.082). All curves decreased with increasing box size as larger windows averaged over local gaps and spatial heterogeneity (Figure 8c). The ranking is closely related to field occupancy: PJ-0 contains a relatively narrow bundle surrounded by extensive background, whereas BPJ-3% occupies a much larger fraction of the frame.

Accordingly, the low lacunarity of BPJ-3% should be interpreted as a more evenly occupied segmented field rather than as evidence of low material porosity. Conversely, the high value for PJ-0 reflects both the background surrounding the bundle and any internal gaps. Lacunarity is therefore useful as a two-dimensional gap-heterogeneity descriptor when interpreted alongside the segmentation matrix, but it does not quantify pore volume, connectivity, or accessibility.

#### 3.6.3. Generalized Dimensions and Singularity Spectra

**Figure 9:**
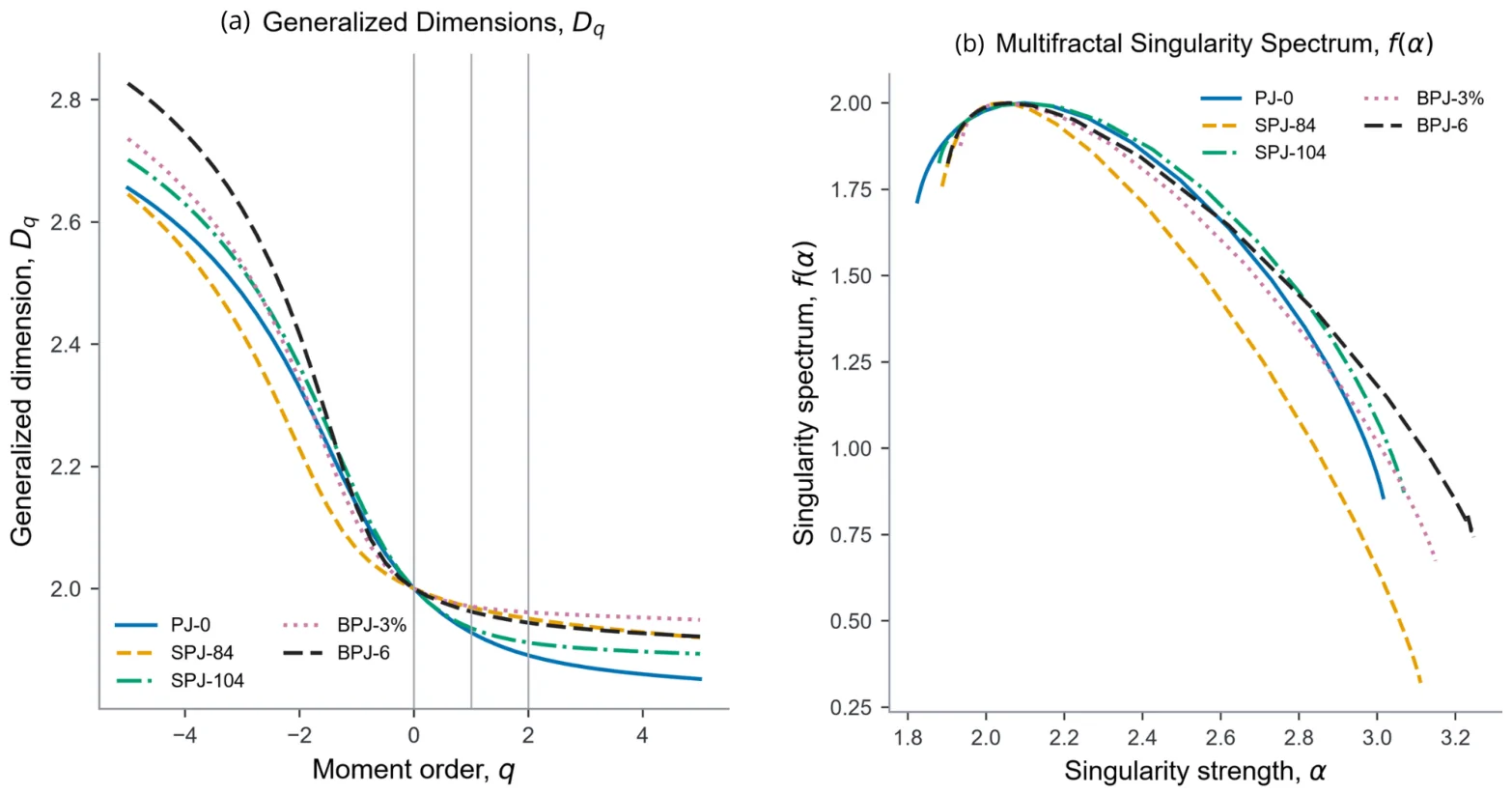
Multifractal analysis of SEM grayscale intensity: (**a**) generalized dimensions $D_{q}$ as a function of moment order *q*; and (**b**) Chhabra–Jensen singularity spectra f(α). The curves describe differences in multiscale grayscale organization among the five processing conditions. Gray solid vertical lines in panel (**a**) mark q=0, q=1, and q=2, corresponding to the capacity dimension $D_{0}$, information dimension $D_{1}$, and correlation dimension $D_{2}$, respectively.

The generalized dimensions and singularity spectra extend the analysis beyond a single global fractal exponent (Figure 9). For all five conditions, $D_{q}$ decreased with increasing *q*, indicating heterogeneous contributions of grayscale intensity across locations and scales. The capacity dimension remained $D_{0}=2.000$ in every case, as expected for the two-dimensional support used in the calculation; this is an internal consistency check, not a measure of the physical dimensionality of the fiber.

BPJ-3% exhibited the largest information and correlation dimensions ($D_{1}=1.9708$ and $D_{2}=1.9612$), whereas PJ-0 showed the lowest values ($D_{1}=1.9280$ and $D_{2}=1.8907$). Within the adopted image measure, this indicates a more even spatial distribution of grayscale intensity in BPJ-3%. The absolute differences are modest and are therefore interpreted together with occupancy, boundary geometry, and the original micrographs rather than as an independent performance ranking.

The singularity-spectrum widths followed a different order: SPJ-104 (1.1886) and PJ-0 (1.1909) were narrowest, BPJ-3% (1.2145) and SPJ-84 (1.2233) were intermediate, and BPJ-6% exhibited the broadest spectrum (1.3444). The selected BPJ-6% field therefore combined the largest nominal boundary dimension with the widest range of grayscale scaling singularities. By contrast, the selected BPJ-3% field combined high $D_{1}$ and $D_{2}$ values with a substantially narrower spectrum, consistent with a more evenly distributed and directionally organized field. The multifractal fits satisfied the predefined internal acceptance criteria, with median scaling $R^{2}$ values close to unity.

### 3.7. Spatial Organization, Spectral Texture, and Robustness

#### 3.7.1. Directional Organization

**Figure 10:**
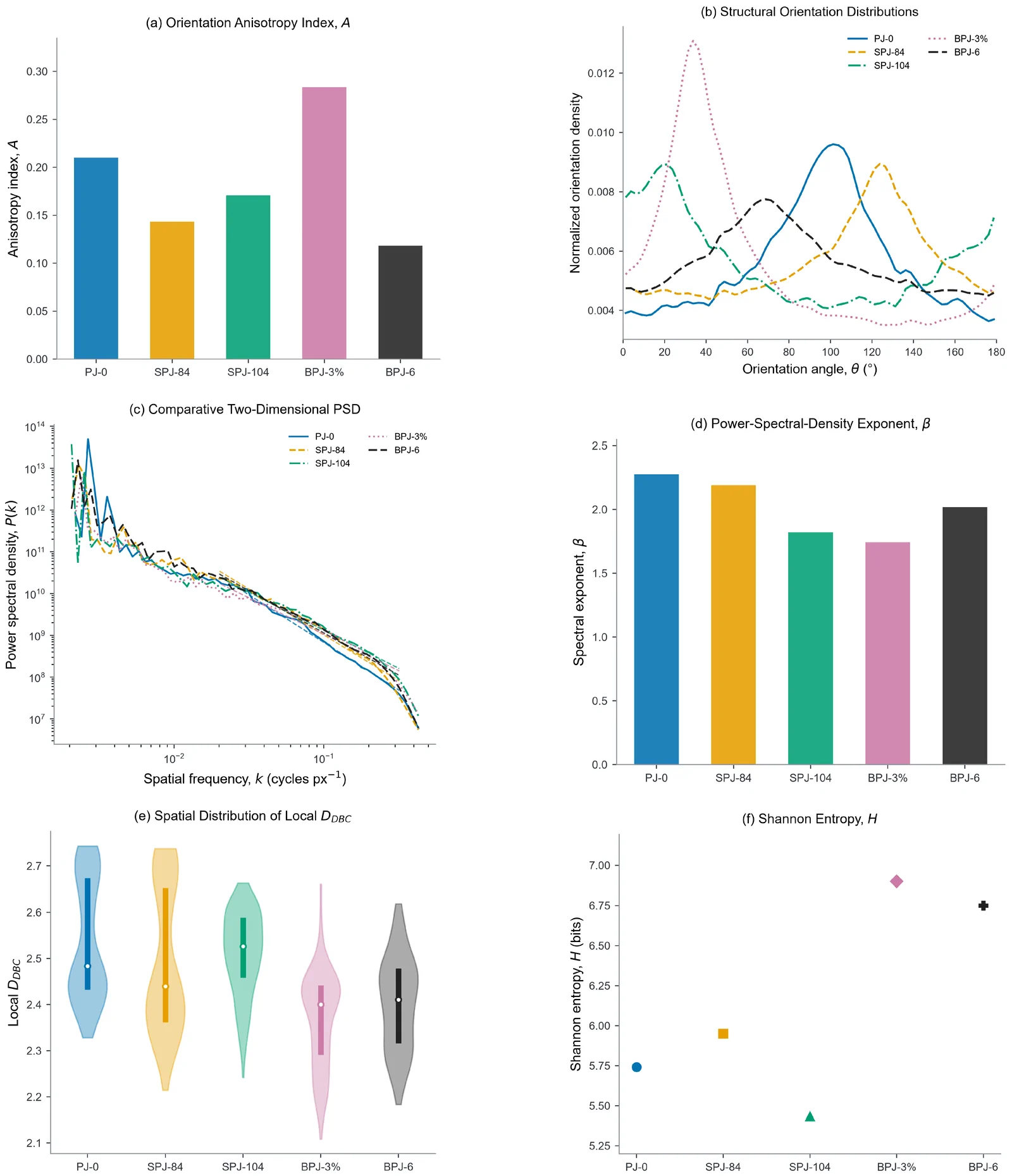
Spatial organization and grayscale texture of the SEM fields: (**a**) axial anisotropy *A*; (**b**) orientation distributions; (**c**) radially averaged power spectral density (PSD); (**d**) spectral exponent β; (**e**) local $D_{\mathrm{DBC}}$ distributions; and (**f**) Shannon entropy *H*. Orientation angles depend on image placement, while panel (**e**) describes within-image variability.

The anisotropy index distinguished directional organization from boundary complexity (Figure 10a; Table 7). The selected BPJ-3% field showed higher axial anisotropy (A=0.2835) than the selected BPJ-6% field (A=0.1183). Across all five selected fields, the numerical values were BPJ-3% (0.2835), PJ-0 (0.2101), SPJ-104 (0.1707), SPJ-84 (0.1434), and BPJ-6% (0.1183). Because only one field was analyzed per condition, this contrast describes the directional organization of the measured fields and does not establish a reproducible treatment-level difference in fiber anisotropy.

The orientation distributions differed in both peak position and width (Figure 10b). The BPJ-3% distribution had a narrow, prominent peak, whereas the BPJ-6% distribution was broader. Because the bundles were not aligned to a common acquisition axis, absolute peak angles are image-coordinate descriptors and cannot be interpreted as treatment-induced physical rotation. The concentration of the distribution, summarized by *A*, therefore provides the more meaningful basis for comparison.

#### 3.7.2. Power Spectral Density and Scale-Dependent Texture

The radially averaged PSD curves decayed approximately according to a power law over the selected fitting interval (Figure 10c), with $R^{2}=0.958$–0.994. The spectral exponent decreased in the order PJ-0 (2.2753) > SPJ-84 (2.1896) > BPJ-6% (2.0182) > SPJ-104 (1.8213) > BPJ-3% (1.7427) (panel d). Within this grayscale framework, higher β values indicate stronger weighting toward coarse spatial organization, whereas lower values reflect a greater relative contribution from finer spatial frequencies.

The selected BPJ-3% field combined higher directional anisotropy with the lowest spectral exponent among the measured fields, indicating a texture in which longitudinal organization coexists with a comparatively strong fine-scale contribution. This differs from the selected BPJ-6% field, which had a more complex boundary network but weaker directional organization. These descriptors characterize different properties of the same measured field; neither boundary dimension nor grayscale scaling alone captures the complete observed morphology. Because low-frequency PSD contributions also reflect overall bundle shape and image framing, the spectra were not converted into fibril-size distributions.

#### 3.7.3. Local Complexity and Intensity Dispersion

Local $D_{\mathrm{DBC}}$ distributions reveal spatial variability that a single whole-image value cannot capture (Figure 10e). The approximate medians were 2.483, 2.439, 2.526, 2.400, and 2.411 for PJ-0, SPJ-84, SPJ-104, BPJ-3%, and BPJ-6%, respectively. SPJ-104 showed the highest local median, whereas the bleached fields exhibited lower medians and broader spatial variation. Because the windows overlap within the same image, these distributions describe local heterogeneity rather than populations of independent replicates.

Shannon entropy was highest for BPJ-3% (6.9006 bits) and BPJ-6% (6.7494 bits), intermediate for SPJ-84 (5.9494 bits) and PJ-0 (5.7413 bits), and lowest for SPJ-104 (5.4338 bits) (panel f). Both bleached fields therefore contained a broader distribution of grayscale states, even though their boundary and orientation metrics differed substantially. Entropy alone cannot distinguish organized surface exposure from fragmentation. Supporting co-occurrence descriptors followed the same general pattern: grayscale contrast was approximately 15.74 and 15.77 for BPJ-3% and BPJ-6%, whereas SPJ-104 showed the highest reported homogeneity (approximately 0.730).

#### 3.7.4. Threshold Sensitivity and Joint Interpretation

Threshold perturbation produced total $D_{B}$ spans of 0.162, 0.231, 0.231, 0.284, and 0.198 for PJ-0, SPJ-84, SPJ-104, BPJ-3%, and BPJ-6%, respectively. These ranges exceed the formal regression standard errors and, for some pairs, are larger than the differences between nominal fitted values. The numerical ordering of $D_{B}$ was not invariant under all plausible threshold offsets, demonstrating that the choice of segmentation is an important source of within-image uncertainty. This sensitivity analysis does not quantify alternative field framing or between-field heterogeneity. The spatial bootstrap results similarly describe variability within the same images and were not interpreted as independent treatment variance.

Because the SEM descriptors are derived from the same individual field, they are not treated as independent lines of statistical evidence. Instead, each descriptor is interpreted according to the image property it represents and in conjunction with the original micrograph and segmentation result. The selected SPJ-104 field showed a comparatively simple contour and low entropy together with a high local grayscale-complexity median. The selected BPJ-3% field showed prominent longitudinal organization, higher axial anisotropy, and high grayscale entropy, whereas the selected BPJ-6% field showed a more irregular boundary network, broader grayscale multifractal organization, and lower directional anisotropy. These differences characterize the individual measured fields and are not interpreted as independently replicated treatment effects.

Studies that link peroxide-induced morphological changes to performance generally pair microscopy with direct measurements of functional properties. For example, Xu et al. combined microstructural characterization of peroxide-treated bamboo with permeability and fungal-resistance tests [34]. Those endpoints were not measured here. The present image analysis instead identifies morphological differences between similarly lightened fibers and relates them to the independent spectroscopic, diffraction, and thermal responses.

### 3.8. Thermogravimetric Response and Thermal Degradation

#### 3.8.1. Principal Degradation Events and Residual Mass

**Figure 11:**
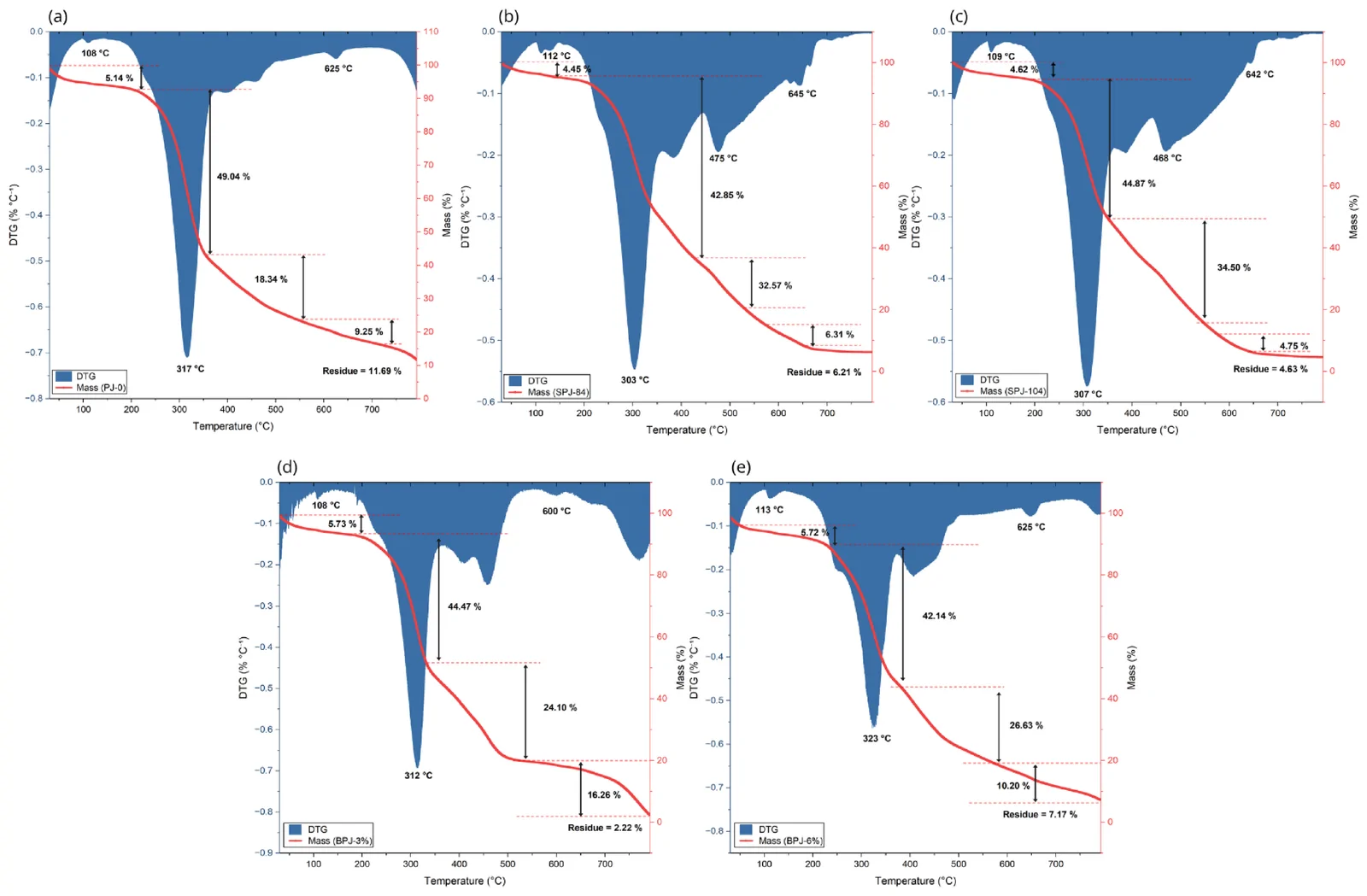
TGA (red) and DTG (blue) profiles of (**a**) PJ-0, (**b**) SPJ-84, (**c**) SPJ-104, (**d**) BPJ-3%, and (**e**) BPJ-6%, recorded under nitrogen at a heating rate of 10 °C min^−1^ up to approximately 800 °C. Dashed guides indicate the temperature intervals used for comparative analysis.

The TGA/DTG profiles display the characteristic multistage degradation of lignocellulosic materials, comprising an initial low-temperature loss, a dominant carbohydrate-rich decomposition region, and broader high-temperature contributions associated with overlapping degradation and char-forming reactions (Figure 11). Because cellulose, hemicellulose, lignin, extractives, and secondary products decompose over overlapping temperature ranges, the individual mass-loss intervals cannot be assigned quantitatively to single constituents [32].

The principal thermal descriptors of the measured specimens are summarized in Table 8.

PJ-0 exhibited a relatively sharp principal degradation event and the highest final residue (11.69%). The dried specimens showed lower $T_{\max}$ values, 303.6 °C for SPJ-84 and 307.2 °C for SPJ-104. The 3.6 °C difference between the SPJ-84 and SPJ-104 profiles is reported descriptively. Given the single-profile design and the lack of independent repeatability estimates, no mechanistic relationship with the XRD-derived apparent coherent-domain dimension is inferred. The SPJ-104 profile also showed a broader post-peak response than SPJ-84, indicating a different distribution of mass-loss rate within the measured profiles.

Among the individual measured TGA/DTG profiles, BPJ-3% reached $T_{\max}=312.8$ °C and retained 2.22% residue, whereas BPJ-6% showed the highest principal degradation-rate temperature of the series ($T_{\max}=322.6$ °C) and 7.17% residue. Because each condition was represented by one thermal profile and no independent repeatability estimate was available, this numerical ordering is interpreted as a characteristic of the measured specimens and not as evidence of a reproducible treatment-level shift in thermal stability. The 9.8 °C difference between the two BPJ profiles therefore remains a specimen-level observation.

This divergence highlights the distinct physical meanings of the measured endpoints. $T_{\max}$ identifies the temperature at which the degradation rate is highest for the surviving solid under the specified heating program; it is not a direct measure of bundle continuity, tensile performance, or service temperature. Likewise, differences in final residue among the measured specimens cannot be interpreted as residual lignin alone because carbonaceous products and native or processing-derived inorganic constituents may contribute.

#### 3.8.2. Common Temperature Windows and Numerical Consistency

Common temperature windows provide a direct cross-sample comparison of how mass loss was redistributed by processing (Table 9). The 30–150 °C mass loss ranged from 4.45 to 5.73%, with both BPJ specimens showing values of approximately 5.7%. This low-temperature interval may include contributions from retained moisture and other volatile species. However, because residual moisture was not independently measured and no controlled post-drying equilibration was applied, differences in this temperature interval are reported descriptively and are not interpreted as quantitative differences in moisture content or moisture accessibility.

The largest mass loss within the common temperature windows occurred between 250 and 380 °C, amounting to 49.04% for PJ-0 and 42.14–44.87% for the laboratory-processed specimens. The dried fibers showed larger contributions between 380 and 600 °C (32.57–34.50%) than the bleached fibers (24.10–26.63%), whereas BPJ-3% showed the largest reported contribution above 600 °C (16.26%) together with the lowest final residue. The measured profiles show that mass loss was redistributed differently across multiple temperature domains.

The sum of the interval mass losses and final residue deviates from 100% by −0.97, −0.38, +0.05, −0.74, and −1.35 percentage points for PJ-0, SPJ-84, SPJ-104, BPJ-3%, and BPJ-6%, respectively. These small departures from mass balance are reported explicitly; the rows were not rescaled to force a 100% balance. Minor differences also exist between selected figure annotations and the tabulated $T_{\max}$ values; numerical comparisons throughout the manuscript use the tabulated values consistently.

### 3.9. Integrated Optical–Structural Interpretation

Taken together, the data reveal distinct specimen-level patterns across the measured processing states. Within the drying comparison, SPJ-84 and SPJ-104 differed in hydroxyl-sensitive FTIR ratios, apparent diffraction descriptors, thermal response, and SEM texture, while their image-derived lightness values showed a different numerical ordering. Dry-state organization, O–H- and water-sensitive spectral descriptors, grayscale texture, and measured appearance therefore showed different numerical orderings across the two dried specimens. Because the principal series contains one independent specimen per condition, these differences cannot be distinguished from between-specimen, acquisition, or processing variability and are not interpreted as evidence of a reproducible treatment-response pattern.

The BPJ-3% and BPJ-6% images showed similar measured appearance in the relative image-based color analysis ($\Delta E_{00}=1.7$), while their selected SEM fields differed in directional organization and boundary characteristics. The selected BPJ-3% field showed higher axial anisotropy and a more concentrated orientation distribution than the selected BPJ-6% field. These comparisons are descriptive and should not be interpreted as evidence that either nominal formulation provides superior whitening, as a formal optimization criterion, or as direct evidence that one treatment preserved the longitudinal fiber architecture.

The selected BPJ-6% field showed a more irregular segmented boundary network, a broader singularity spectrum, and lower directional anisotropy than the selected BPJ-3% field, while the individual BPJ-6% TGA/DTG profile showed a higher $T_{\max}$. In the individual measured XRD profiles, BPJ-3% showed higher apparent Scherrer and Segal descriptors than BPJ-6%. The XRD and TGA/DTG profiles therefore provide complementary specimen-level characterization but do not independently establish a reproducible treatment trend because each processing condition is represented by a single profile.

Importantly, the SPJ-104-to-BPJ comparison represents the complete alkaline-oxidative processing sequence. Hydrogen peroxide was introduced within an alkaline medium, and the BPJ specimens were also subjected to renewed wetting, heating, washing to neutral pH, and redrying. These coupled chemical and processing steps can simultaneously modify swelling, accessibility, non-cellulosic environments, surface organization, and residual volatile content. In the absence of chemistry-resolved and matched processing controls, the differences observed relative to SPJ-104 are therefore interpreted as the integrated response of the BPJ sequence rather than as a peroxide-specific effect.

The SEM comparison is likewise restricted to the morphology of the selected fields. Differences in occupancy, field framing, segmentation threshold, and unmeasured between-field heterogeneity limit the extent to which boundary-complexity or anisotropy rankings can be generalized to the corresponding processing conditions.

The condition with the higher nominal peroxide-stock addition should not be considered intrinsically inferior for every application. Extensive fibrillation is deliberately pursued in micro- and nanofibrillated cellulose production [13], whereas maintenance of a coherent bundle represents a different processing objective. Likewise, the suitability of *Carludovica palmata* fibers as reinforcement cannot be inferred without direct composite-property measurements [16]. The present conclusions therefore concern the observed specimen states rather than validated end-use performance.

### 3.10. Scope and Limitations of the Comparison

The central limitation of the principal physicochemical series is the absence of independent treatment and specimen replication. With one independent specimen per processing state, the study cannot estimate biological variability, variability between specimens, or variability in treatment preparation. Nor can it establish whether the observed differences are reproducible across independent specimens or batches. The results are therefore interpreted as comparative observations of the measured specimen states rather than as population-level treatment estimates.

The external validity of the present study is necessarily limited by the sampling design. All principal specimens originated from a single artisan source and batch; the numbers of individual source plants and parent fiber bundles were not recorded, and one independent specimen was characterized per processing condition. In addition, PJ-0 had already undergone the traditional artisan-processing sequence before the laboratory experiments and therefore represents a pre-processed technological reference rather than pristine botanical tissue or an untreated biological control.

The interpretation is additionally constrained by the absence of separate alkali-only, peroxide-only, and matched wash/redry controls. Image-derived CIELAB values are not equivalent to calibrated reflectance measurements, and several SEM descriptors remain sensitive to segmentation, field occupancy, and framing. Micro-ROIs, overlapping windows, scaling fits, regression errors, threshold perturbations, and bootstrap resamples improve characterization of the measured images but do not increase the number of independent treatment preparations or provide treatment-level uncertainty.

Consequently, the measured descriptor values and their numerical ordering should be interpreted as characteristics of the investigated specimen states and should not be generalized to *Carludovica palmata* fibers from other plants, harvests, artisan batches, geographic origins, or processing histories without independent replication across these levels. Mechanistic interpretations involving hydration, packing, oxidation, or modification of non-cellulosic domains are presented as hypotheses consistent with the measured profiles. No claims are made regarding molecular weight, tensile performance, three-dimensional porosity, or end-use reinforcement behavior.

## 4. Conclusions

Comparative multiscale characterization revealed differences in the optical, supramolecular, spectroscopic, morphological, and thermal descriptors among the measured processing states of artisan-processed *Carludovica palmata* fibers, while the principal cellulose-associated diffraction features and the polysaccharide-rich ATR-FTIR fingerprint remained present. These observations indicate that the characteristic cellulose-rich lignocellulosic framework persisted across the investigated specimen states despite differences detected by the complementary analytical techniques.

The BPJ-3% and BPJ-6% specimen images showed similar measured appearance in the relative image-based color analysis ($\Delta E_{00}=1.7$), whereas their structural descriptors differed. The selected BPJ-3% SEM field exhibited greater directional organization, and its individual XRD profile showed higher apparent Segal and Scherrer descriptors. In contrast, the selected BPJ-6% field showed lower anisotropy and a more irregular boundary network, while its individual TGA/DTG profile exhibited the highest principal degradation-rate temperature of the series. Within the measured specimens, these results show that similar image-derived appearance can coexist with different morphological, diffraction, and thermal descriptors.

Overall, the study highlights the value of combining complementary multiscale techniques when characterizing processed lignocellulosic fibers, since no single descriptor represented the complete physicochemical response. Because the principal series contained one independent specimen per condition and originated from a single artisan batch, these findings are interpreted as characteristics of the measured specimen states rather than reproducible treatment-level effects or evidence of an optimized processing condition.

## Figures and Tables

**Table 1:** Processing conditions and experimental contrasts.

Sample	Precursor and Conditioning	Additional Bath Treatment	Drying After the Stated Step
PJ-0	Artisan-processed reference	No laboratory bleaching	Artisanal processing history
SPJ-84	Fiber conditioned in water, 54 °C, 1 h	None	84 °C, 1 h; time-defined endpoint
SPJ-104	Fiber conditioned in water, 54 °C, 1 h	None	104 °C, 1 h; time-defined endpoint
BPJ-3%	SPJ-104	1.60 g NaOH + 4.0 mL peroxide stock; 40 mL final volume; 70 °C, 1 h	60 °C, 5 h after washing
BPJ-6%	SPJ-104	1.60 g NaOH + 8.0 mL peroxide stock; 40 mL final volume; 70 °C, 1 h	60 °C, 5 h after washing

**Table 2:** Operational fraction recovery reported as mean ± standard deviation from three independent fractionation experiments. Fraction names denote operationally enriched materials rather than chemically pure components. Because the procedure did not provide a closed analytical mass balance, the reported recoveries should not be interpreted as quantitative constituent percentages.

Operational Fraction	Mass (g)	Recovery (%)	Normalized Share (%)
Cellulose-enriched	0.6790±0.0517	67.90±5.17	68.04±1.96
Hemicellulose-enriched	0.2686±0.0172	26.86±1.72	26.94±1.15
Lignin-enriched	0.0524±0.0354	5.24±3.54	5.03±3.09

**Table 3:** Relative image-derived CIELAB descriptors for one parent ROI per condition. Neither independently acquired standardized color measurements nor estimates of between-specimen color variability were available. Values are therefore used only for internal comparison of the analyzed images and do not represent instrumentally calibrated reflectance or standardized whiteness measurements.

Sample	$L^{*}$	$a^{*}$	$b^{*}$	$\Delta E_{00}$ vs. PJ-0
PJ-0	62	−1.27	27.71	0
SPJ-84	75	−2.65	23.98	10.3
SPJ-104	72	−1.46	21.54	8.5
BPJ-3%	86	−0.80	9.23	20.6
BPJ-6%	84	−0.61	11.08	19.0

**Table 4:** Apparent XRD descriptors derived from one measured diffraction profile per condition. $D_{\mathrm{app}}$ denotes the apparent Scherrer dimension and ${\mathrm{CrI}}_{\mathrm{app}}$ the apparent Segal crystallinity index. No independent replicate profiles were available; therefore, the reported values represent specimen-level observations rather than treatment-level estimates.

Sample	2θ (°)	FWHM (°)	*d* (Å)	$D_{\mathrm{app}}$ (nm)	${\mathrm{CrI}}_{\mathrm{app}}$ (%)
PJ-0	22.230	3.121	3.996	2.71	85.8
SPJ-84	22.145	3.486	4.011	2.43	74.5
SPJ-104	22.593	3.389	3.932	2.50	75.8
BPJ-3%	22.143	3.195	4.011	2.65	81.2
BPJ-6%	22.037	3.410	4.030	2.48	78.9

**Table 5:** ATR-FTIR absorbance ratios normalized to the 1030 cm^−1^ polysaccharide band.

Ratio	PJ-0	SPJ-84	SPJ-104	BPJ-3%	BPJ-6%
$A_{\mathrm{OH}}/A_{1030}$	0.147	0.221	0.124	0.234	0.285
$A_{1620}/A_{1030}$	0.603	0.695	0.604	0.845	0.802
$A_{1730}/A_{1030}$	0.205	0.272	0.196	0.396	0.247
$A_{1235}/A_{1030}$	0.227	0.203	0.228	0.204	0.178
$A_{1160}/A_{1030}$	0.234	0.280	0.218	0.342	0.238

**Table 6:** Fractal and multifractal descriptors of the individual SEM fields. Each row represents one SEM field from one condition. The ± values are regression standard errors of the fitted scaling relations and do not represent between-specimen or treatment-level uncertainty. $\bar{\Lambda }$ is the mean lacunarity; Δα is the singularity-spectrum width. $D_{0}=2.000$ for all five image measures.

Sample	$D_{B}$	$D_{\mathrm{DBC}}$	$\bar{\Lambda }$	$D_{1}$	$D_{2}$	Δα
PJ-0	1.3877±0.0125	2.4540±0.0340	6.330	1.9280	1.8907	1.1909
SPJ-84	1.4667±0.0034	2.4720±0.0439	3.615	1.9693	1.9509	1.2233
SPJ-104	1.3219±0.0128	2.4987±0.0402	4.611	1.9352	1.9116	1.1886
BPJ-3%	1.3643±0.0055	2.4747±0.0444	2.082	1.9708	1.9612	1.2145
BPJ-6%	1.5570±0.0022	2.4840±0.0449	2.801	1.9623	1.9441	1.3444

**Table 7:** Spatial-texture descriptors and segmentation sensitivity of the SEM fields. Local $D_{\mathrm{DBC}}$ values are reported as medians, and the $D_{B}$ span represents the variation produced by threshold changes.

Sample	Foreground (%)	*A*	β	*H* (bits)	Local $D_{\mathrm{DBC}}$ Median	$D_{B}$ Threshold Span
PJ-0	12.3	0.2101	2.2753	5.7413	2.483	0.162
SPJ-84	21.7	0.1434	2.1896	5.9494	2.439	0.231
SPJ-104	18.1	0.1707	1.8213	5.4338	2.526	0.231
BPJ-3%	42.7	0.2835	1.7427	6.9006	2.400	0.284
BPJ-6%	26.3	0.1183	2.0182	6.7494	2.411	0.198

**Table 8:** Thermal descriptors derived from one TGA/DTG profile per condition. DTG is the derivative of normalized residual mass with respect to temperature, and residue is the remaining mass near 793 °C under nitrogen. No independent replicate profiles or repeatability estimates were available; the values are therefore reported as observations of the measured specimens.

Sample	$T_{\max}$ (°C)	DTG Minimum (% °C^−1^)	Mass at $T_{\max}$ (%)	Residue (%)
PJ-0	316.0	−0.710	62.02	11.69
SPJ-84	303.6	−0.547	68.57	6.21
SPJ-104	307.2	−0.574	67.47	4.63
BPJ-3%	312.8	−0.693	63.10	2.22
BPJ-6%	322.6	−0.563	63.00	7.17

**Table 9:** Mass loss across selected temperature intervals and final pyrolytic residue. All values are expressed as percentages.

Sample	30–150 °C	150–250 °C	250–380 °C	380–600 °C	600–793 °C	Residue	Sum
PJ-0	5.14	5.57	49.04	18.34	9.25	11.69	99.03
SPJ-84	4.45	7.23	42.85	32.57	6.31	6.21	99.62
SPJ-104	4.62	6.68	44.87	34.50	4.75	4.63	100.05
BPJ-3%	5.73	6.48	44.47	24.10	16.26	2.22	99.26
BPJ-6%	5.72	6.79	42.14	26.63	10.20	7.17	98.65

## Data Availability

The data supporting the findings of this study are provided within the article and its Supplementary Materials.

## Supplementary Materials

The following supporting information can be downloaded at https://www.mdpi.com/article/10.3390/polym18202507/s1, Figure S1: Sensitivity of boundary fractal dimension to segmentation-threshold perturbation for the five analyzed SEM fields; Figure S2: Individual boundary box-counting fits for PJ-0, SPJ-84, SPJ-104, BPJ-3%, and BPJ-6%; Figure S3: Spatial maps of local differential-box-counting fractal dimension $D_{\mathrm{DBC}}$ for the five selected SEM fields; Figure S4: Orientation distributions and power-spectral-density (PSD) curves for the five selected SEM fields; Figure S5: Multifractal diagnostics for the five selected SEM grayscale fields; Table S1: Sensitivity of boundary fractal dimension to segmentation-threshold perturbation for the five selected SEM fields; Table S2: Diagnostics of the boundary box-counting regressions used to estimate $D_{B}$ in the five selected SEM fields; Table S3: Complementary spatial-texture descriptors for one selected SEM field per condition; Table S4: Multifractal descriptors calculated from the positive grayscale-intensity measure for one selected SEM field per condition; Table S5: Essential acquisition, processing, and interpretation parameters used for the principal image-based and instrumental analyses.
