# Supplementary material for: Multiscale Physicochemical Response of Lignocellulosic Carludovica palmata Fibers to Controlled Drying and Alkaline Hydrogen Peroxide Treatment
Source: Polymers (Basel). 2026 Oct 13;18(20):2507. doi: 10.3390/polym18202507 (PMC13709129; doi:10.3390/polym18202507)
Supplement: Supplementary file 1 [file polymers-18-02507-s001.zip › polymers-4598653-supplementary.pdf]

Supplementary Materials: Multiscale Physicochemical Response of Lignocellulosic *Carludovica palmata* Fibers to Controlled Drying and Alkaline Hydrogen Peroxide Treatment

Manuel Ayala-Chauvin [1,*] and Sebastian Ojeda [1]

S1. Supplementary SEM Image-Analysis Results

The supplementary analyses reported here correspond to the same five selected SEM fields evaluated in the main manuscript: PJ-0, SPJ-84, SPJ-104, BPJ-3%, and BPJ-6%. One SEM field was analyzed for each condition, and segmented foreground occupancy ranged from 12.3% to 42.7% across the selected images. Accordingly, the analyses presented below provide diagnostics of model fit, segmentation sensitivity, spatial heterogeneity, directional organization, and multiscale grayscale structure within the individual measured fields. They do not represent independent SEM fields, independent specimens, or treatment-level replication.

The supplementary descriptors are therefore interpreted according to the image property represented by each metric and in conjunction with the original micrographs and segmentation outcomes. Agreement among multiple descriptors derived from the same field is not treated as additional experimental replication or as independent statistical evidence.

S1.1. Sensitivity of Boundary Fractal Dimension to Segmentation Threshold

Boundary fractal dimension (D_B) was recalculated after perturbing the nominal segmentation threshold from −20 to +20 grayscale levels in increments of 5 (Figure S1). Across the nine thresholds evaluated for each image, the total D_B span ranged from 0.1621 to 0.2841 (Table S1). The numerical ordering of D_B was not invariant to all plausible threshold offsets, indicating that the calculated boundary-fractal descriptor depends on the adopted segmentation.

Foreground occupancy is reported together with the threshold-sensitivity statistics because the amount and spatial distribution of segmented foreground, as well as image framing, can influence boundary-based descriptors. The statistics in Table S1 therefore quantify sensitivity of the individual image analysis to threshold selection and should not be interpreted as between-specimen or treatment-level uncertainty.

Table S1. Sensitivity of boundary fractal dimension to segmentation-threshold perturbation for the five selected SEM fields. Foreground occupancy is included as contextual information on segmented image content. Mean, standard deviation (SD), coefficient of variation (CV), and total span were calculated across nine thresholds ranging from −20 to +20 grayscale levels relative to the nominal threshold. These statistics describe within-image processing sensitivity and are not estimates of treatment-level uncertainty.

Sample	Foreground occupancy (%)	Nominal threshold	D_B	Mean D_B	SD D_B	CV (%)	Total span
PJ-0	12.3	110	1.3877	1.3803	0.0554	4.01	0.1621
SPJ-84	21.7	108	1.4667	1.4852	0.0906	6.10	0.2306
SPJ-104	18.1	98	1.3219	1.3201	0.0832	6.30	0.2306
BPJ-3%	42.7	90	1.3643	1.3654	0.1039	7.61	0.2841
BPJ-6%	26.3	104	1.5570	1.4901	0.0714	4.79	0.1976

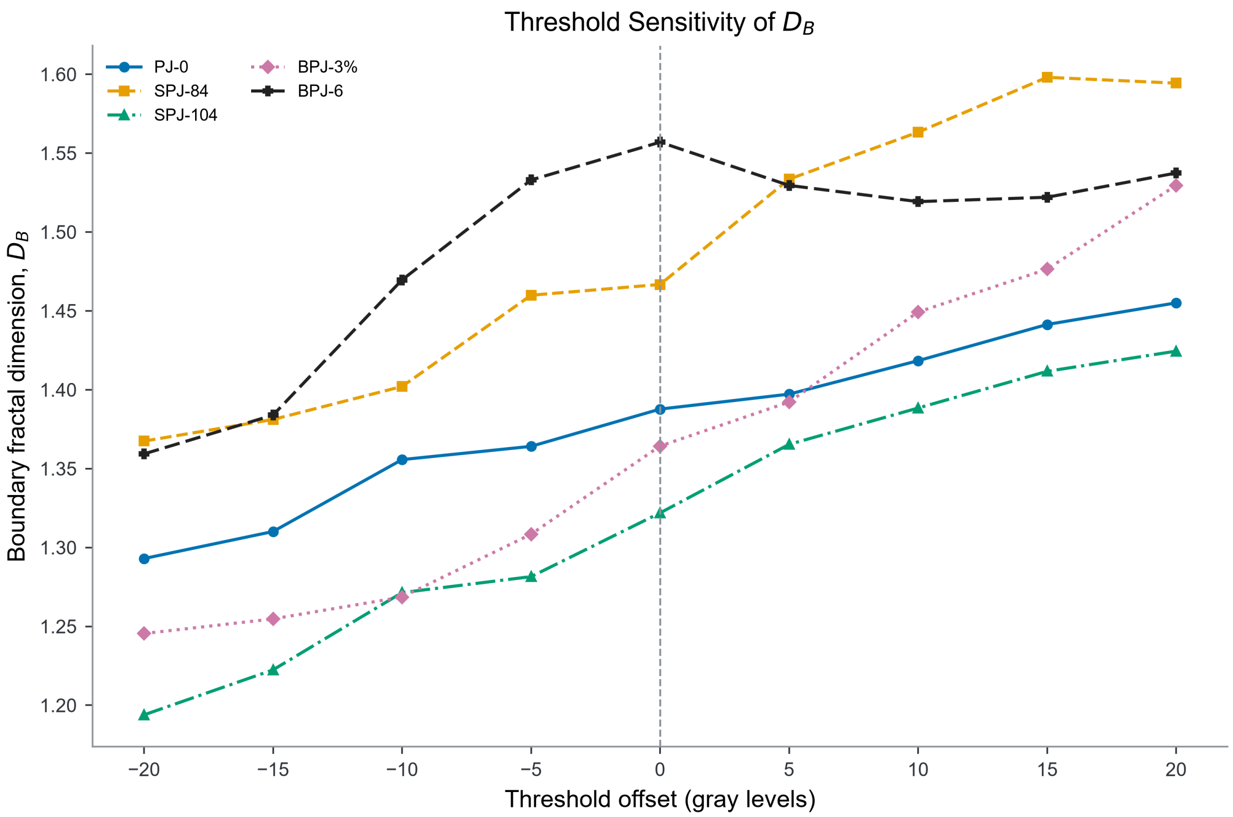

Figure S1. Sensitivity of boundary fractal dimension to segmentation-threshold perturbation for the five analyzed SEM fields. Alternative threshold offsets were applied to the same image field for each condition. The resulting variation, including changes in the numerical ordering of D_B under some threshold offsets, illustrates the dependence of the boundary-fractal descriptor on segmentation. This analysis characterizes within-image processing sensitivity and does not represent independent replication or treatment-level uncertainty.

S1.2. Boundary Box-Counting Regression Diagnostics

Individual translated-grid box-counting regressions for the selected fields showed high linearity over the retained scaling windows (Figure S2). Each fit used 23 effective scales spanning approximately 2.08–2.10 decades, with R^2 values between 0.9980 and 1.0000 (Table S2).

The regression standard errors and confidence intervals quantify uncertainty in the fitted slope of the scaling relation for each individual image. They do not capture sensitivity to segmentation threshold, alternative field selection, between-specimen variation, or treatment-level uncertainty.

Table S2. Diagnostics of the boundary box-counting regressions used to estimate D_B in the five selected SEM fields. SE is the regression standard error of the fitted slope, and CI is the 95% regression confidence interval. These quantities describe fit uncertainty for the individual image and do not estimate between-field, between-specimen, or treatment-level uncertainty.

Sample	D_B	SE	95% CI	R^2	Included scales	Scale span (decades)
PJ-0	1.3877	0.0125	[1.3616, 1.4138]	0.9983	23/23	2.079
SPJ-84	1.4667	0.0034	[1.4596, 1.4739]	0.9999	23/23	2.099
SPJ-104	1.3219	0.0128	[1.2953, 1.3485]	0.9980	23/23	2.104
BPJ-3%	1.3643	0.0055	[1.3530, 1.3756]	0.9997	23/23	2.079
BPJ-6%	1.5570	0.0022	[1.5523, 1.5616]	1.0000	23/23	2.104

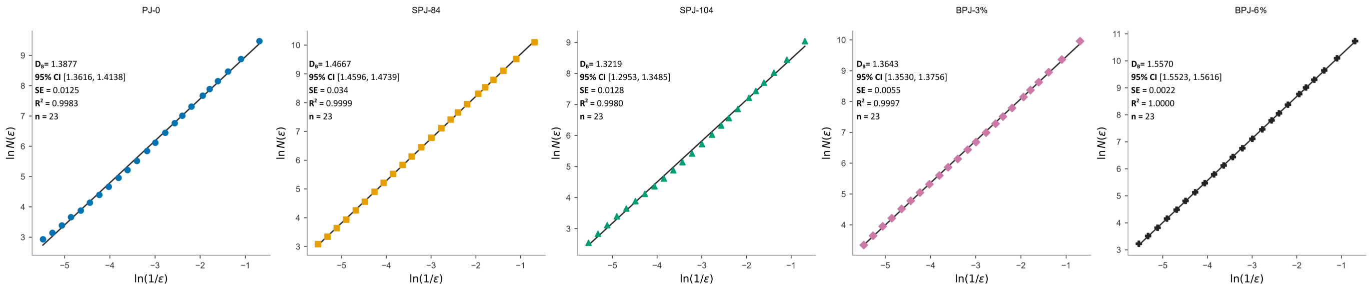

Figure S2. Individual boundary box-counting fits for PJ-0, SPJ-84, SPJ-104, BPJ-3%, and BPJ-6%. Symbols represent $\ln N(\varepsilon)$ as a function of $\ln(1/\varepsilon)$, and solid lines indicate the selected linear scaling windows. The fitted slope corresponds to the boundary fractal dimension D_B.

S1.3. Spatial Heterogeneity of Local Grayscale Complexity

Local differential-box-counting analysis was used to examine spatial variation in grayscale complexity that is not represented by a single whole-image D_{DBC} value (Figure S3). Approximate median local values were 2.483, 2.439, 2.526, 2.400, and 2.411 for PJ-0, SPJ-84, SPJ-104, BPJ-3%, and BPJ-6%, respectively.

The selected SPJ-104 field illustrates the complementary nature of the SEM descriptors: it showed the lowest nominal boundary dimension ($D_B = 1.3219$) but the highest median local grayscale-complexity value among the five analyzed fields. Boundary geometry and grayscale-intensity complexity therefore describe different properties of the same image. Because the local analysis was performed using overlapping windows within a single field, the resulting maps describe within-image heterogeneity only.

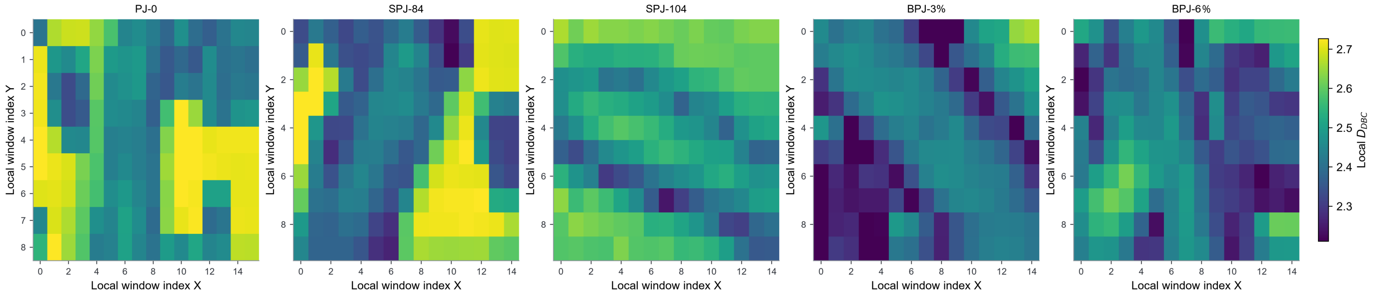

Figure S3. Spatial maps of local differential-box-counting fractal dimension D_{DBC} for the five selected SEM fields. Higher values indicate greater local multiscale variation in grayscale intensity. A common color scale is used to facilitate comparison among the displayed fields. The maps represent within-image spatial heterogeneity and do not constitute independent experimental replicates.

S1.4. Directional Organization and Power-Spectral Diagnostics

Orientation distributions and radially averaged power spectral densities (PSD) for the five selected SEM fields are presented in Figure S4. The BPJ-3% field showed the highest axial anisotropy among the selected fields ($A = 0.2835$), whereas the BPJ-6% field showed the lowest ($A = 0.1183$).

Because the fiber bundles were not registered to a common longitudinal axis before imaging, the absolute dominant angles are descriptors of the coordinate system of each image and are not directly compared as common physical directions. The rotation-invariant anisotropy magnitude A is therefore used to describe the degree of directional organization within the measured field.

The PSD fits yielded power-law exponents of $\beta = 1.7427$–2.2753 with $R^2 = 0.958$–0.994 (Table S3). These descriptors characterize spatial-frequency content within each selected image and are not interpreted as independently replicated treatment effects.

Table S3. Complementary spatial-texture descriptors for one selected SEM field per condition. A is axial orientation anisotropy, θ is the dominant image-coordinate orientation, β is the PSD power-law exponent, and H is Shannon entropy. Local D_{DBC} values are approximate medians of the within-image maps. These descriptors characterize the individual measured fields and are not independent experimental replicates.

Sample	A	θ (°)	β	PSD R^2	H (bits)	Local D_{DBC}
PJ-0	0.2101	97.98	2.2753	0.994	5.7413	2.483
SPJ-84	0.1434	123.11	2.1896	0.977	5.9494	2.439
SPJ-104	0.1707	15.61	1.8213	0.972	5.4338	2.526
BPJ-3%	0.2835	36.13	1.7427	0.958	6.9006	2.400
BPJ-6%	0.1183	71.06	2.0182	0.985	6.7494	2.411

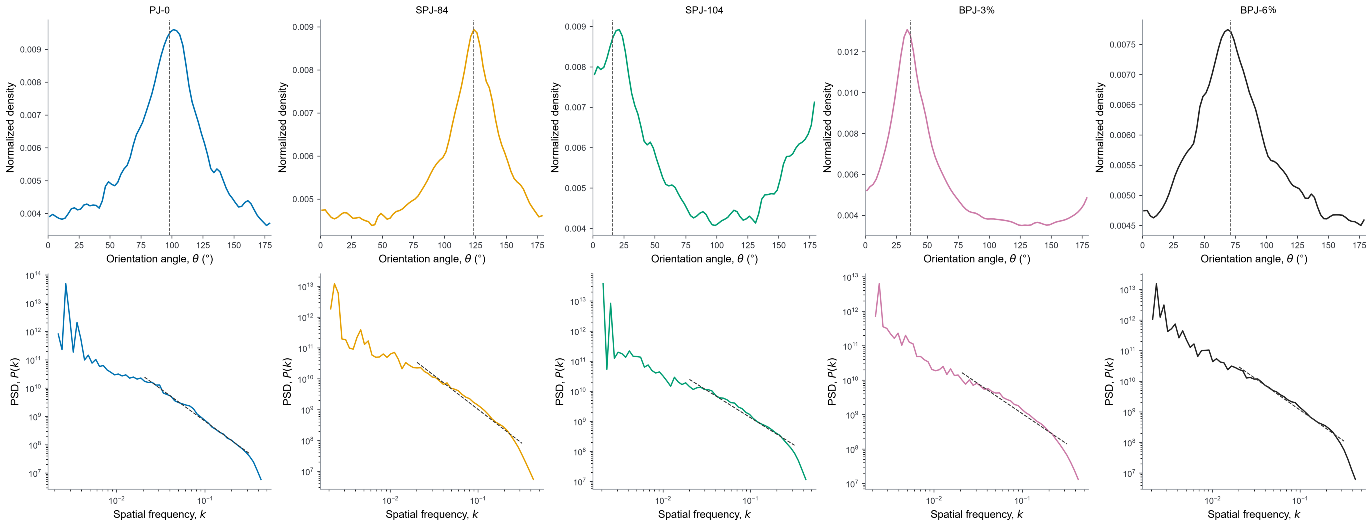

Figure S4. Orientation distributions and power-spectral-density (PSD) curves for the five selected SEM fields. Top row: normalized axial orientation distributions over 0–180°; dashed vertical lines indicate the dominant orientation in the coordinate system of each image. Bottom row: radially averaged PSD as a function of spatial frequency; dashed segments indicate the interval used to fit the relation $P(k) \propto k^{-\beta}$. Absolute dominant angles are image-coordinate descriptors because the fields were not registered to a common longitudinal axis.

S1.5. Generalized Dimensions and Chhabra–Jensen Singularity Spectra

Generalized dimensions decreased with increasing moment order q in all five selected grayscale fields, with $D_0 = 2.000$ (Figure S5). The singularity-spectrum widths $\Delta\alpha$ were 1.1909, 1.2233, 1.1886, 1.2145, and 1.3444 for PJ-0, SPJ-84, SPJ-104, BPJ-3%, and BPJ-6%, respectively (Table S4).

Among the individual measured fields, BPJ-6% showed the broadest singularity spectrum ($\Delta\alpha = 1.3444$), whereas BPJ-3% showed the highest D_1 and D_2 values. At the nominal segmentation threshold, the BPJ-6% field also showed the highest numerical D_B. These descriptors represent different aspects of grayscale and boundary organization and are interpreted as descriptive properties of the individual fields rather than as independent evidence of reproducible treatment-level differences.

S2. Supplementary Interpretation and Scope

The supplementary analyses provide additional characterization of the same individual SEM fields reported in the main manuscript and clarify the sensitivity and scope of the corresponding image-derived descriptors.

The selected BPJ-3% field showed higher axial anisotropy ($A = 0.2835$), whereas the selected BPJ-6% field showed lower anisotropy ($A = 0.1183$), a more irregular segmented boundary network at the nominal threshold, and the broadest singularity spectrum ($\Delta\alpha =$

Table S4. Multifractal descriptors calculated from the positive grayscale-intensity measure for one selected SEM field per condition. D_0, D_1, and D_2 are the capacity, information, and correlation dimensions, respectively; $\Delta\alpha$ is the singularity-spectrum width. The values characterize the individual measured fields and do not constitute independent treatment replication.

Sample	D_0	D_1	D_2	$\Delta\alpha$	Median R^2
PJ-0	2.0000	1.9280	1.8907	1.1909	0.9992
SPJ-84	2.0000	1.9693	1.9509	1.2233	1.0000
SPJ-104	2.0000	1.9352	1.9116	1.1886	0.9994
BPJ-3%	2.0000	1.9708	1.9612	1.2145	1.0000
BPJ-6%	2.0000	1.9623	1.9441	1.3444	1.0000

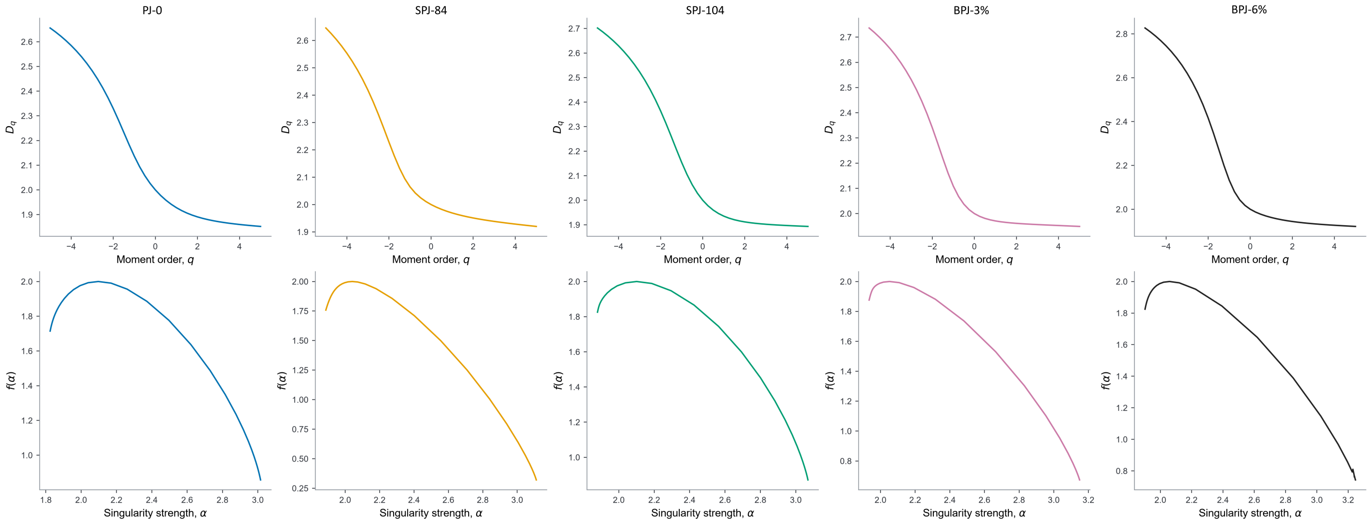

Figure S5. Multifractal diagnostics for the five selected SEM grayscale fields. Top row: generalized dimensions D_q over the moment-order interval $q = -5$ to 5. Bottom row: direct Chhabra–Jensen singularity spectra $f(\alpha)$ as a function of singularity strength α. All curves were derived from the individual grayscale field corresponding to each condition.

1.3444). The selected SPJ-104 field further illustrates that the descriptors represent different image properties, combining a comparatively low nominal D_B with the highest median local D_{DBC} among the five analyzed fields.

The threshold-sensitivity analysis showed that segmentation-induced variation in D_B exceeded the formal regression-fit error for several images and that the numerical ordering of D_B changed under some plausible threshold perturbations. Boundary-fractal values are therefore interpreted together with foreground occupancy, segmentation behavior, and the original SEM field rather than as a stand-alone treatment ranking.

More generally, all SEM descriptors in this supplementary file originate from one selected field per condition. Agreement among boundary, grayscale, multifractal, spectral, or directional descriptors derived from the same image does not increase the number of independent experimental units. Differences in field occupancy, framing, segmentation threshold, and unmeasured between-field heterogeneity limit generalization beyond the individual fields analyzed.

S3. Supplementary Reproducibility Information

Figures S1–S5 and Tables S1–S5 provide the diagnostic results and essential processing information required to interpret the SEM image analyses reported in the main manuscript.

No separate numerical XRD data package is provided with the Supplementary Information; the XRD results and corresponding analytical information are reported within the article and its Supplementary Materials.

No separate numerical XRD data package is provided with the Supplementary Information; the XRD results and corresponding analytical information are reported within the article and its Supplementary Materials.

Table S5. Essential acquisition, processing, and interpretation parameters used for the principal image-based and instrumental analyses.

Analysis or step	Essential parameters
Sampling hierarchy	One condition-level specimen for each of PJ-0, SPJ-84, SPJ-104, BPJ-3%, and BPJ-6%; no independently prepared replicate treatment baths or independently treated replicate specimens in the principal physicochemical series. One SEM field was analyzed per condition. Within-image resampling and model-fitting procedures were not treated as independent experimental replication.
SPJ processing	Fiber portions conditioned in water at 54 °C for 1 h and subsequently dried for 1 h at either 84 or 104 °C. The drying endpoint was defined by the prescribed 1 h duration. No controlled post-drying temperature/relative- humidity conditioning was applied, and residual moisture was not independently measured.
BPJ bath formulation	SPJ-104 used as precursor. Each bath contained 1.60 g NaOH and either 4.0 or 8.0 mL of peroxide stock labeled 30%, with distilled water added to a final volume of 40 mL. BPJ-3% and BPJ-6% are retained as nominal treatment labels because the concentration basis of the commercial peroxide stock was not recorded.
BPJ processing	Treatment at 70 °C for 1 h with magnetic stirring at 350 rpm, followed by 5–6 water washes to neutral pH and final drying at 60 °C for 5 h. No subsequent controlled temperature/relative-humidity conditioning was applied, and residual moisture was not independently measured before the physicochemical analyses.
SEM acquisition	Backscattered-electron imaging in BSD Full mode; 2000× magnification; 259 μm field width; 10 kV accelerating voltage; 60 Pa chamber pressure; working distance approximately 8 mm; one field analyzed per condition; spatial calibration from the embedded 50 μm scale bar.
SEM preprocessing and segmentation	Microscope footer excluded; robust intensity normalization using the 0.5th–99.5th percentile range; Otsu thresholding with Li thresholding as a fallback when the Otsu threshold approached an extreme of the occupied intensity range; bright- and dark-foreground alternatives evaluated automatically; small isolated objects and holes removed; 3 × 3 morphological-gradient edge network extracted from the retained segmentation.

Analysis or step	Essential parameters
Boundary fractal analysis	Translated-grid box counting; 16 translated grid origins; 24 logarithmically distributed target scales from 2 pixels to one quarter of the minimum image dimension; duplicate integer scales removed; contiguous scaling windows containing at least five scales evaluated for linearity, scale span, and local-slope stability.
SEM threshold sensitivity	D_B recalculated at threshold offsets from -20 to $+20$ grayscale levels in increments of 5. The analysis quantifies within-image segmentation sensitivity and does not estimate between-field or treatment-level uncertainty.
Spatial bootstrap analysis	Two hundred spatial bootstrap resamples used to summarize within-image heterogeneity. Bootstrap resamples were not treated as independent experimental replicates.
SEM orientation analysis	Gaussian smoothing with $\sigma = 1$ pixel followed by Sobel differentiation; axial orientation evaluated over 0–180°; anisotropy calculated from the rotation-invariant axial orientation magnitude. Absolute dominant angles were treated as image-coordinate descriptors because fields were not registered to a common longitudinal axis.
SEM spectral texture	Radially averaged two-dimensional PSD calculated after mean subtraction and application of a two-dimensional Hann window; power-law fitting performed over 0.02–0.35 cycles pixel^{-1}; Shannon entropy calculated from the 8-bit intensity histogram.
Multifractal analysis	Direct Chhabra–Jensen analysis of the positive grayscale-intensity measure; largest centered power-of-two square analyzed without interpolation; exact dyadic box sizes; 41 moment orders from $q = -5$ to 5; D_q, $\alpha(q)$, and $f(\alpha)$ retained when the stated regression-quality criteria were satisfied.
Relative image-based color analysis	One parent ROI per condition with radius 200 pixels; 500 pseudo-random micro-ROIs of radius 4 pixels; fixed random seed 20260911; sRGB linearization; conversion to CIE XYZ using a D65 computational reference followed by CIE 1976 $L^*a^*b^*$. No certified color target or instrument-traceable reflectance reference was available; the resulting color coordinates and ΔE_{00} values were therefore interpreted as relative image-derived descriptors.
XRD acquisition	Rigaku MiniFlex 600 with D/teX Ultra2 detector; $2\theta = 5$–50°; sampling interval 0.01°; Cu Kα radiation, $\lambda = 1.5406$ Å; one measured comparative profile per condition.

Analysis or step	Essential parameters
XRD processing	Profiles processed in Profex using a SNIP background with decreasing clipping window and 120 iterations. The estimated background was subtracted from each original diffraction profile to obtain a baseline-corrected pattern, followed by a 25-point simple moving average corresponding to 0.25° in 2θ. Peak positions and FWHM values were obtained using the QualX peak-search routine. Scherrer calculations used $K = 0.94$. No separate crystalline line-broadening reference standard was measured, and no explicit instrumental-broadening correction was applied. No separate multi-peak deconvolution or Rietveld refinement was used to obtain the reported apparent Scherrer dimensions.
ATR-FTIR	PerkinElmer Spectrum Two FT-IR with PIKE MIRacle UATR accessory; three scans; 4000–650 cm^{-1}; transmittance converted to absorbance using $A = -\log_{10}(T/100)$; baseline correction applied consistently. Spectral ratios were interpreted as relative descriptors and not as quantitative measurements of polymer composition or residual moisture.
TGA/DTG	TA Instruments TGA55; 100 μL platinum pans; nitrogen atmosphere; heating to approximately 800 °C at 10 °C min^{-1}; DTG expressed as dW/dT; one measured thermal profile per condition. The 30–150 °C interval was treated descriptively because residual moisture was not independently measured.
